# Color‐Filter‐Free Image Sensor Using CsPbBr_3_ Quantum‐Dot‐Based Tamm Plasmon Photodetector for Photonic Synapse Facial Recognition

**DOI:** 10.1002/advs.202503464

**Published:** 2025-06-25

**Authors:** Meng‐Cheng Yen, Yung‐Chi Yao, Chia‐Jung Lee, Shi‐Hui Huang, Wei Jie Hong, Yuto Kajino, Hsu‐Cheng Hsu, Min‐Hsiung Shih, Gong‐Ru Lin, Kaoru Tamada, Jinn‐Kong Sheu, Ya‐Ju Lee

**Affiliations:** ^1^ Graduate Institute of Photonics and Optoelectronics Department of Electrical Engineering National Taiwan University (NTU) No. 1, Sec. 4, Roosevelt Road Taipei 10617 Taiwan; ^2^ Program on Key Materials, Academy of Innovative Semiconductor and Sustainable Manufacturing (AISSM) National Cheng Kung University No. 1, University Road Tainan City 70101 Taiwan; ^3^ Department of Photonics National Cheng Kung University No. 1, University Road Tainan City 70101 Taiwan; ^4^ Institute for Materials Chemistry and Engineering (IMCE) Kyushu University 744 Motooka, Nishiku Fukuoka 819‐0395 Japan; ^5^ Research Center for Applied Sciences Academia Sinica Taipei 11529 Taiwan

**Keywords:** CsPbBr_3_, image sensor, neuromorphic computing, photodetector, quantum dots, Tamm plasmon

## Abstract

This study demonstrates that optical absorption of the CsPbBr_3_ quantum dots can be significantly enhanced through monolithic integration with the Tamm plasmon (TP) structure. This integration enables the resulting TP photodetector to achieve a higher photocurrent and a more linear power dependence compared to the reference device with a nonresonant configuration. The enhancement is confined to the designed resonant energy, while photons with off‐resonance energies are fully reflected, making the TP photodetector an ideal candidate for compact and efficient image sensors, eliminating the need for additional filters or bulky microlenses for color discrimination or photon collection. Furthermore, the photocurrent generated by the TP photodetector can be regulated by varying light pulse stimulations, enabling it to mimic the synaptic dynamics of the human brain. By integrating the functions of perception, processing, and memorization of visual images, its potential for facial recognition through simulation based on a 64 × 64 array of TP photodetectors under the artificial neural network‐based weight‐update expectation thresholding model is demonstrated. These findings mark a significant step forward in utilizing all‐inorganic perovskite materials for compact, color‐filter‐free image sensors and open new avenues for photonic neural computations on a single platform.

## Graphical abstract

A cutting‐edge color‐filter‐free photodetector that seamlessly integrates a monolayer perovskite quantum dots absorber by leveraging Tamm plasmon resonance enhances optical absorption through strong light–matter coupling and demonstrates synaptic‐like dynamics, enabling neuron‐inspired facial recognition.

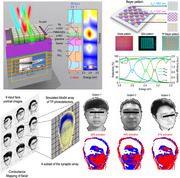

## 1 Introduction

In recent years, image sensors have experienced significant advancements, leading to their widespread utilization across a broad range of applications, from high‐end cameras in media production to ubiquitous devices such as smartphones, Internet of Things (IoT) gadgets, and security and surveillance systems.^[^ ^1^, ^2^, ^3^, ^4^, ^5^ ^]^ At the core of the image sensor lies the photodetector, a vital component that absorbs incident photons and converts them into electrical charges proportional to light intensity. Depending on the underlying technology, such as complementary metal‐oxide‐semiconductor (CMOS)^[^ ^6^, ^7^ ^]^ or charge‐coupled device (CCD),^[^ ^8^, ^9^ ^]^ these electrical charges are either directly converted into voltage at each photosite or transferred to adjacent capacitors for further processing. The resulting voltage or charge is then digitized by an analog‐to‐digital converter and processed by integrated circuitry to produce a digital image of the captured scene. By nature, photodetectors respond only to the total number of photogenerated carriers, initially producing monochromatic images without color information. To enable color imaging, a color filter array made of organic dye‐based absorption material is added to the sensor.^[^ ^10^ ^]^ However, these filters have inherent limitations: they degrade under high temperatures or prolonged ultraviolet exposure^[^ ^11^ ^]^ and cannot be thinner than a few hundred nanometers due to the dye's low absorption coefficient.^[^ ^12^ ^]^ In addition, microlenses are typically placed over each photosite to focus incoming light onto the photodetector, enhancing photon collection by reducing optical losses caused by surface reflections.^[^ ^13^ ^]^ As pixel size in image sensors continues to shrink, integrating color filter arrays and microlenses presents challenges in compatibility with standard photodetector fabrication processes and cost‐effective manufacturing techniques.^[^ ^14^ ^]^

To this end, the rising demand for advanced image sensors has driven extensive research toward novel functional materials that offer not only superior photodetection capabilities but also provide greater control over light‐matter interactions compared to traditional semiconductors like Si, Ge, and InGaAs.^[^ ^15^, ^16^, ^17^, ^18^, ^19^, ^20^, ^21^ ^]^ Among these emerging candidates, colloidal quantum dots (QDs) with dimensions approaching or below their exciton Bohr radius, such as lead chalcogenides (e.g., PbS, PbSe)^[^ ^22^, ^23^ ^]^ and II–VI semiconductors (e.g., CdSe, HgTe),^[^ ^24^, ^25^ ^]^ have demonstrated great potential for broadband photodetection applications. More recently, low‐dimensional all‐inorganic perovskite QDs have garnered significant attention, with various synthesis methods being developed to optimize their performance.^[^ ^26^, ^27^ ^]^ The interest in perovskite QDs stems from their tunable optical properties, particularly their emission and absorption spectra, which can be precisely controlled through adjustments to their dimensionality or morphology, leveraging quantum confinement effects.^[^ ^28^, ^29^, ^30^ ^]^ In addition, all‐inorganic perovskites exhibit excellent resistance to environmental degradation, making them well‐suited for practical applications.^[^ ^31^, ^32^ ^]^ For instance, our group recently developed a neuromorphic vision system that seamlessly integrates optical sensing and synaptic functionalities into a unified material platform, exclusively employing CsPbBr_3_ QDs synthesized via a supersaturated recrystallization process.^[^ ^33^ ^]^ In a related study, Shen et al. developed flexible photodetector arrays based on CsPbBr_3_ QDs, utilizing CsBr/KBr surface treatment to enhance surface morphology and crystallinity, thereby reducing defect density and promoting photogenerated carrier extraction.^[^ ^18^ ^]^ Wang et al. improved the performance of CsPbBr_3_ QD‐based photodetectors by modifying ligands and incorporating poly(3‐hexylthiophene) (P3HT),^[^ ^20^ ^]^ while Zhao et al. introduced a transparent photodetector featuring an active layer of CsPbBr_3_ QDs, layered between a poly‐TPD organic blend and a composite dielectric made of polymethyl methacrylate (PMMA) and polyacrylic acid (PAA).^[^ ^21^ ^]^ Despite these advancements, most research on CsPbBr_3_ QD‐based photodetectors has concentrated on atomic‐level ligand techniques, such as ligand density control and exchange, to increase the internal quantum yield of CsPbBr_3_ QDs. However, these methods often involve high‐temperature processing or intricate synthesis procedures, posing challenges for streamlined and cost‐effective device fabrication. As a result, there is a growing need for alternative approaches that improve the photodetection capabilities of CsPbBr_3_ QD‐based photodetectors through external structural integration. These strategies not only have the potential to enhance the optical absorption of CsPbBr_3_ QDs but also eliminate the need for additional components, such as color filters and bulky optical lenses, leading to more compact and efficient image sensors.

In this study, we report on an advanced photodetector design by integrating the TP structure with an all‐inorganic CsPbBr_3_ QDs absorber. The TP, analogous to Tamm electronic surface states in semiconductors, is a confined electromagnetic eigenmode localized at the interface between an isotropic medium with a negative dielectric constant (e.g., a normal metal below its plasma frequency) and a distributed Bragg reflector (DBR).^[^ ^27^, ^34^, ^35^ ^]^ Unlike conventional surface plasmons, the TP's dispersion lies within the light cone defined by *k*  =  ω/*c*, where *k* is the in‐plane wave vector component, and ω is the angular frequency. This allows for direct excitation of the TP eigenmode in a planar configuration using optical excitation with both TE and TM polarizations. In addition, the CsPbBr_3_ QDs are strategically placed at the peak field of the TP eigenmode. When the incident light resonates with the frequency of the TP eigenmode, the confined electromagnetic field strengthens photon‐exciton coupling, leading to greater absorption and higher photocurrent in the TP photodetector. In contrast, off‐resonance light is fully reflected, suppressing the device's photoresponse at noninteractive optical frequencies. This selective resonance mechanism provides enhanced photodetection with exceptional color or wavelength selectivity, eliminating the need for additional color filters or bulky microlenses.^[^ ^36^ ^]^ Furthermore, the TP photodetector exhibits a nonvolatile photocurrent behavior, attributed to the inclusion of the PMMA layer that creates a conduction band discontinuity and effectively traps photogenerated electrons.^[^ ^37^, ^38^ ^]^ The photocurrent can be regulated by varying the light pulse stimulations, showing neuromorphic synaptic plasticity features such as excitatory postsynaptic current (EPSC), paired‐pulse facilitation (PPF), and long‐term potentiation/depression (LTP/LTD). Consistent with the neuromorphic characteristics observed in previously reported photonic synapses,^[^ ^39^, ^40^ ^]^ these intelligent functionalities make the TP photodetector highly suitable for mimicking the human visual system. To demonstrate its potential, we further simulated a 64 × 64 array of TP photodetectors as photonic neural synapses to emulate facial recognition. This demonstration underlines the TP photodetector's unique combination of enhanced color‐selective photodetection and neuromorphic computing capabilities.

## 2 Results and Discussion

### 2.1 Design of CsPbBr_3_ QD‐Based Tamm Plasmon Photodetector

**Figure 1:**
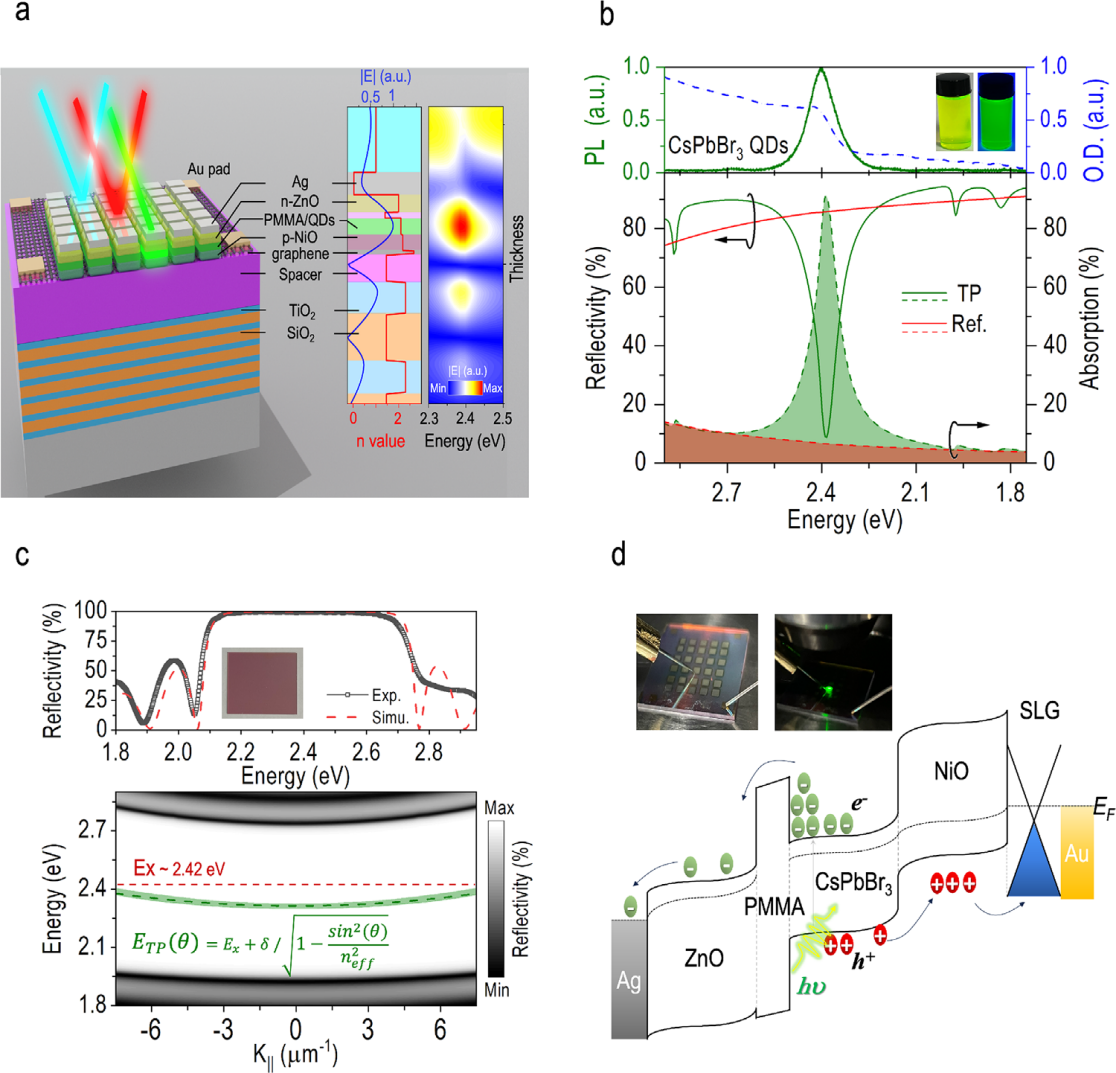
CsPbBr_3_ QD‐based Tamm‐plasmon (TP) photodetector with enhanced optical absorption at specific resonant eigenmode. a) Schematic illustration of the TP photodetector, designed to enhance optical absorption at a specific resonant eigenmode through strong light–matter interactions. This design efficiently reflects off‐resonance photons without requiring additional color filters (left). The electric field intensity distribution inside the TP photodetector at its resonant energy of *E_TP_* = 2.38 eV, along with the refractive index (*n*) profile along the device's normal axis (middle). Simulations of spatial and spectral *E* distributions across the TP photodetector (right). b) PL and absorption spectra of the as‐synthesized CsPbBr_3_ QDs (top panel), accompanied by a photograph under UV (right inset) and normal (left inset) lighting conditions. Simulated reflectivity (solid lines) and absorption (dashed lines, calculated as *A*  =  1 − *R* − *T*) spectra for both the reference device and the TP photodetector (bottom panel). The resonant energy of TP eigenmode is precisely aligned with the excitonic absorption peak of CsPbBr_3_ QDs to promote photon–exciton interactions. c) Measured and simulated reflectance spectra of the DBR bottom reflector (top panel), demonstrating high coherence. The inset shows a photograph of the DBR viewed at a normal angle, revealing its reddish appearance. Simulated momentum‐resolved reflectivity spectra of the TP photodetector (bottom panel), showing a sharp, dispersive eigenmode within the photonic stopband of the DBR. d) Schematic energy diagram of the TP photodetector, illustrating the separation of photogenerated electron‐hole pairs under a negative electrical bias. The voltage is applied to the top Ag electrode, while the single‐layer graphene attached to the Au pad is grounded (appearing green color when the incident light is focused on the device, see the photograph inset in the figure).

**Figure** **1**a illustrates the schematic configuration of the CsPbBr_3_ QD‐based TP photodetector (left panel). This device is meticulously engineered to optimize optical absorption at a specific incident wavelength through strong light–matter interactions, while efficiently reflecting off‐resonance photons. The nominal resonant energy of the TP eigenmode (*E*_TP_) is set to *E*_TP_ = 2.38 eV (corresponding to an optical wavelength of λ  =  520 nm), matching the emission energy of the CsPbBr_3_ QDs and showing a Stokes shift of ≈ 40 meV relative to their excitonic absorption peak at 2.42 eV (top plot, Figure 1b). The device also allows for broad tunability of the resonant energy, covering wavelengths from ultraviolet to deep green, provided that the wide absorption spectrum of CsPbBr_3_ QDs and the adjustable TP eigenmode align with the respective wavelength. Simulations of spatial and spectral distributions of electric field (*E*) across the device confirm the exclusive excitation of a single resonant eigenmode at ≈ 2.38 *eV* (right panel, Figure 1a). This resonant energy can be precisely controlled by altering the thickness of the SiO_2_ spacer layer in the bottom reflector or by adjusting the number of monolayers in the CsPbBr_3_ QDs absorber, as discussed later in the article. The TP eigenmode forms a standing wave due to the interference between the counter‐propagating incident and reflected light waves, with its amplitude peaking right at the location of the CsPbBr_3_ QDs absorber (middle panel, Figure 1a). As shown in the bottom plot of Figure 1b, the simulated reflectivity spectrum of the TP photodetector (green line) exhibits a pronounced dip down to *R* ≈ 8.7% with a quality factor of *Q* ≈ 62, corresponding to the TP eigenmode. In contrast, the reference device, which lacks the resonant cavity, does not display this feature and instead shows a planar reflectivity characteristic as a typical Ag mirror (red line). The absorption (*A*) at 2.38 eV, calculated as *A*  =  1 − *R* − *T* (where *T* represents transmission), is estimated to be *A*  =  6.58% for the reference device (red dashed line) and *A*  =  91.27% for the TP photodetector (green dashed line), reflecting a 14‐fold enhancement. This improvement is primarily attributed to the strong confinement of the TP eigenmode, which strengthens photon–exciton coupling and causes more energy to be absorbed.^[^ ^41^, ^42^ ^]^

In the TP photodetector, the bottom reflector is a distributed Bragg mirror (DBR) made up of 10 alternating pairs of TiO_2_ and SiO_2_ dielectric layers (see Figure S1, Supporting Information), which provided a high reflectivity of *R*  =  98.30% over a spectral range from 2.13 to 2.66 eV, consistent with the numerical estimation (top plot, Figure 1c). The DBR's photonic stopband reflects optical wavelengths between 460 and 605 nm in the visible‐light spectrum, giving it a reddish appearance, as seen in the photograph inserted in the figure. Unlike previous designs that relied on nonconductive, all‐dielectric DBRs to construct microcavities,^[^ ^15^, ^43^ ^]^ this study uses metallic silver (Ag) as the top mirror. Ag was selected for its two key advantages: (1) its high reflectivity and low damping losses in the visible spectrum, facilitating the efficient excitation of the TP resonance with high finesse, and (2) its ability to serve as an Ohmic contact for the photodetector. Here shall be addressed that the use of a DBR as the bottom mirror in this work is not indispensable. Replacing both the top and bottom mirrors with conductive reflectors, such as in a metal/semiconductor/metal (MSM) stack configuration,^[^ ^44^ ^]^ could enhance CMOS compatibility by enabling vias‐based interconnects for serial modulation of the photogenerated current. However, the inherently higher optical losses associated with metallic reflectors broaden the absorption spectrum and lower the quality factor, both of which compromise the spectral and color selectivity of the photodetector. Furthermore, the MSM stack supports Fabry–Pérot resonances instead of TP modes, imposing stricter constraints on the cavity length and necessitating precise control of the absorber thickness to meet the resonance conditions.^[^ ^45^ ^]^ In addition, several materials‐related challenges, such as the toxicity of lead and the incompatibility of cesium with standard CMOS fabrication processes, must be addressed before the TP devices can be feasibly integrated into CMOS platforms.

The CsPbBr_3_ QDs, synthesized via the hot‐injection method, are utilized as the light‐absorbing layer sandwiched between the Ag mirror and the DBR. The insets of Figure 1b show photographs of the as‐synthesized CsPbBr_3_ QDs under UV illumination (right) and normal lighting conditions (left). To efficiently extract photogenerated carriers, zinc oxide (ZnO) and nickel oxide (NiO) are employed as electron and hole transport layers, respectively, placed above and below the CsPbBr_3_ QDs absorber. A single monolayer graphene (SLG) is transferred onto the DBR using wet chemical processes. Positioned adjacent to the NiO layer, it functions as a spreading layer to enhance the flow of photogenerated currents. In the simulation, the SLG is modeled with a complex refractive index expressed as $\tilde{n}\left(\lambda \right)=3.0+i\left(C_{1}/3\right)\lambda$, where *C*_1_ = 5.446 µm^−1^ and λ is the wavelength.^[^ ^46^ ^]^ Despite the high real part of $\tilde{n}\left(\lambda \right)$, the minimal thickness of the SLG ensures its negligible effect on the TP eigenmode distribution. As a result, a distinct energy state corresponding to the TP eigenmode appears within the photonic stopband (bottom plot, Figure 1c) across a wide range of incident angles (θ) or in‐plane momenta (*k*_||_), following the relation *k*_||_ = *k* · sin(θ), where *k* is the momentum of the incident photon. The dependence of *E_TP_* on θ is expressed as:^[^ ^47^ ^]^

(1) $$E_{TP}\left(\theta \right)=\left(E_{x}+\delta \right)/\sqrt{1-\left(sin^{2}\left(\theta \right)/n_{eff}^{2}\right)}$$

where *E_x_* is the excitonic absorption peak of the CsPbBr_3_ QDs, δ is the exciton–photon detuning, and *n_eff_* is the effective refract index of the TP photodetector. For this study, these parameters are set as *E_x_* = 2.42 eV, δ  =   −37 meV, and *n_eff_* = 1.92. Equation (1) shows that the TP eigenmode can be excited even at large θ, enhancing the detector's practicality for daily applications. In addition, a PMMA layer was intentionally spin‐coated between the CsPbBr_3_ QDs and the ZnO layer. As illustrated in the energy‐band diagram in Figure 1d, which outlines the conduction band edge (*E_c_*), valence band edge (*E_V_*), and Fermi level (*E_f_*) for each component of the TP photodetector (determined through ultraviolet photoelectron spectroscopy, see Figure S2, Supporting Information), the PMMA layer introduces a discontinuity at *E_c_* with a barrier height of ϕ_*B*_ = 1.4 eV. This barrier traps and accumulates photogenerated electrons, allowing the TP photodetector to emulate synaptic dynamics under light pulse stimulation, as will be discussed later. Moreover, the EPSC response is significantly influenced by the thickness of the PMMA layer, which is controllable by adjusting the concentration of PMMA in toluene during the spin‐coating process (see Figure S3, Supporting Information). In this study, a PMMA concentration of 1.5 wt% is utilized, as it provides an optimal trade‐off between photoresponsivity and potentiation retention, both of which are essential for the effective emulation of biological synaptic functionalities. The current–voltage (*I–V*) measurements were conducted using a microscope system. The objective lens was utilized to focus the incident light onto the Ag mirror, which was electrically connected to a probe serving as the bias electrode. Meanwhile, a second probe was positioned on the Au pad situated at the periphery of the device, which lies atop the SLG, and was electrically grounded. These two probes together constitute a closed‐loop electrical circuit for collecting the photogenerated current, as illustrated in the inset images of Figure 1d. More details about the synthesis of CsPbBr_3_ QDs, SLG transfer, device fabrication, and measurement of the TP photodetector, as well as related electromagnetic wave simulations, can be found in the experimental section.

**Figure 2:**
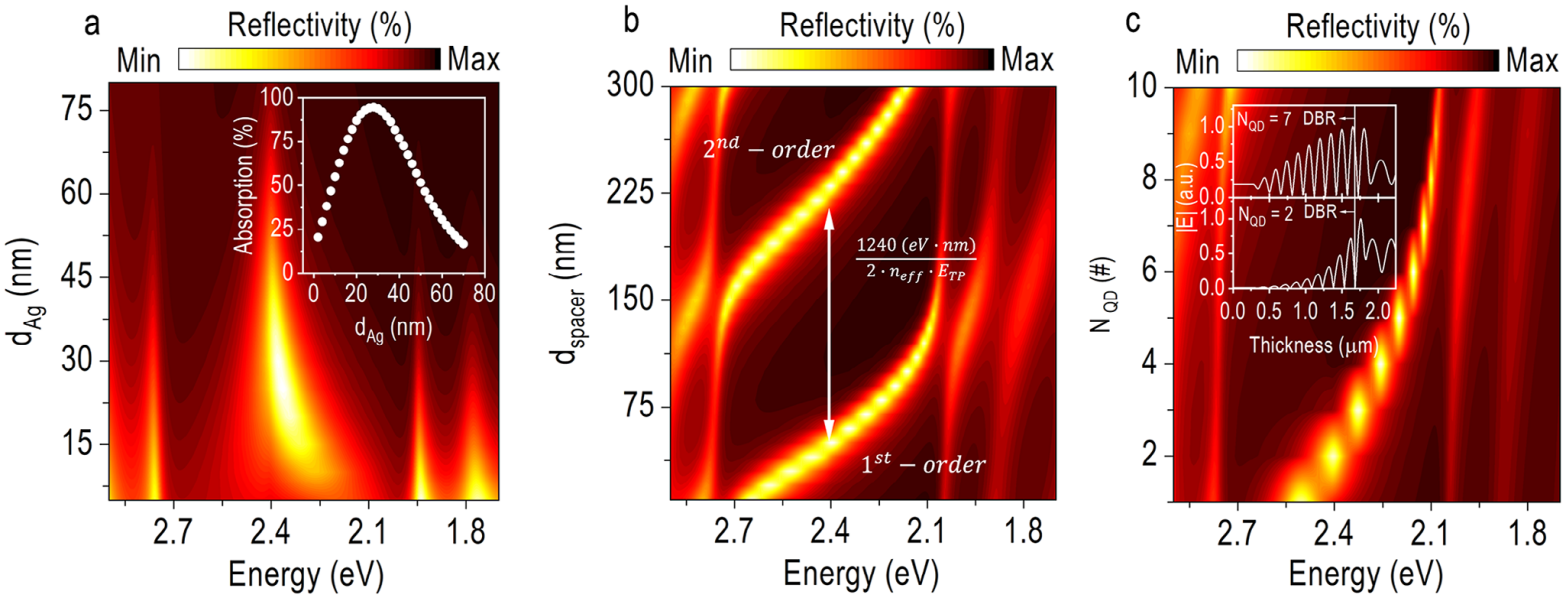
Tuning resonant eigenmode in the CsPbBr_3_ QD‐based TP photodetector. The reflectivity of the TP photodetector is analyzed using finite element method (FEM) simulations performed with COMSOL Multiphysics as a function of the a) thickness of the top Ag mirror (*d_Ag_*), b) thickness of the SiO_2_ spacer layer in the bottom DBR reflector (*d_spacer_*), and c) number of monolayer in the CsPbBr_3_ QDs absorber (*N_QD_*). The inset of (Figure a) shows the simulated optical absorption of the TP photodetector at *E_TP_* = 2.38 eV as a function of *d_Ag_*. In addition, (Figure c) includes the spatial distribution of the E‐field at *E_TP_* = 2.38 eV for the TP photodetector with *N_QD_* = 7 (top inset) and *N_QD_* = 2 (bottom inset), also obtained from FEM simulations.

Building on the confirmation of the successful excitation of TP eigenmode in our photodetector device, we now turn our focus on exploring its tunability. This investigation aims to demonstrate the feasibility of developing color‐filter‐free TP photodetectors that can be tailored to specific photon energies as needed. **Figure** **2** shows how *E_TP_* varies with three key parameters of the TP photodetector, including (a) the thickness of the top Ag mirror (*d_Ag_*), (b) the thickness of the SiO_2_ spacer layer in the bottom DBR reflector (*d_spacer_*), and (c) the number of monolayer in the CsPbBr_3_ QDs absorber (*N_QD_*). A clear bright spot, indicative of the excitation of the TP eigenmode, is observed within the photonic stopband of the DBR (Figure 2a). The thickness of *d_Ag_* directly influences the intensity of the TP eigenmode, as it significantly affects the device's dip reflectivity and quality factor. When *d_Ag_* is thinner than 15 nm, the Ag mirror lacks sufficient reflectivity, leading to optical leakage as light escapes out instead of being trapped between the Ag and DBR reflectors. Conversely, if *d_Ag_* exceeds 60 nm, most incident light is reflected before entering the system, hindering effective TP eigenmode excitation. Thus, optimizing *d_Ag_* is crucial to balancing efficient excitation with minimal reflectivity losses, with an optimal thickness of ≈30 nm yielding the highest absorption for the TP photodetector (inset, Figure 2a). In addition, *E_TP_* varies continuously with *d_spacer_*, allowing precise tuning to any energy within the DBR's photonic stopband (Figure 2b). However, this tuning is nonlinear due to the inherent dispersion behavior of TP polaritons. As *d_spacer_* increases, higher‐order TP eigenmodes appear. For a given *E_TP_*, the interval of *d_spacer_* required to excite two adjacent degenerate TP eigenmodes can be expressed as $d_{spacer}\left(interval\right)=\frac{1240(eV\cdot nm)}{2n_{eff}\cdot E_{TP}}$.^[^ ^48^ ^]^ Finally, as *N_QD_* increases, *E_TP_* decreases, and the bright spot becomes narrower, indicating a gradual suppression of the TP eigenmode (Figure 2c). The spatial distribution analysis of the *E*‐field confirms that when *N_QD_* exceeds seven monolayers, inefficient confinement of the TP eigenmode occurs, resulting in its leakage into the bottom DBR reflector (inset, Figure 2c). This phenomenon is attributed to the phase change induced by the thick CsPbBr_3_ QDs absorber, which disrupts the equilibrium condition for the formation of TP eigenmode. By optimizing these structural parameters, the spectral response of the TP photodetector can be tailored to achieve the desired selectivity without relying on conventional color filters. In this study, the fabricated TP photodetector used *d_Ag_*, *d_spacer_* and *N_QD_* values of 40 nm, 50 nm, and two monolayers, respectively. This specific design ensures that the TP resonance energy is closely aligned with the excitonic absorption peak of the CsPbBr_3_ QDs (i.e., *E_TP_* ≈ 2.42 eV), thereby facilitating strong photon–exciton coupling and substantially enhancing optical absorption. Meanwhile, the chosen Ag mirror thickness (*d_Ag_* = 40 nm) maintains a reasonably low contact resistance (≈5.9 Ω, as estimated in Figure S4, Supporting Information), which is essential for efficient extraction of photogenerated carriers. The fabricated TP photodetector shows a small device‐to‐device variation in the resonant energy of the TP eigenmode and the associated reflectivity dip, demonstrating a high level of uniformity across the array configuration (see Figure S5, Supporting Information). It is worth noting that, for the long‐term pursuit of vertically integrating the TP photodetector with CMOS devices, the SiO_2_ spacer layer may be substituted with alternative polymers such as PVK or PEDOT:PSS. These materials possess comparable refractive indices with SiO_2_ in the visible spectrum^[^ ^49^, ^50^ ^]^ and exhibit p‐type conductive characteristics,^[^ ^51^ ^]^ thereby offering potential advantages for both optical and electrical integration.

### 2.2 Optical Characteristics and Structural Analysis of CsPbBr_3_ QD‐Based TP Photodetector

**Figure 3:**
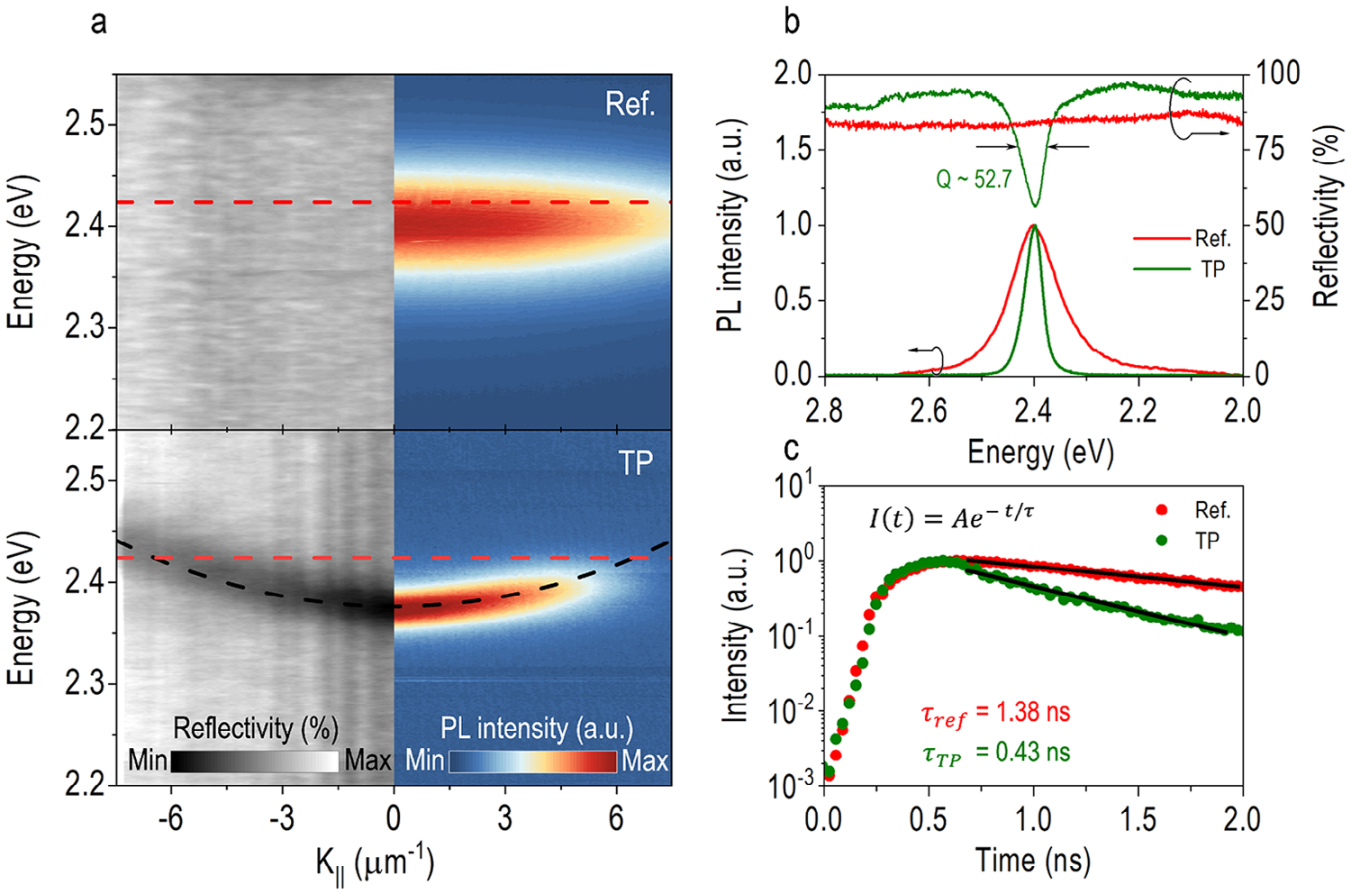
Dispersion relation and photon lifetime of the CsPbBr_3_ QD‐based TP photodetector. a) Momentum‐resolved PL (right) and reflectivity (left) spectra for the reference device (top) and the TP photodetector (bottom). b) Spectral response comparison between the reference device (red lines) and the TP photodetector (green lines), including both PL (primary vertical axis) and reflectivity spectra (secondary vertical axis). The reference device exhibits a relatively flat reflection profile with an average value of *R* ≈ 83.1%, whereas the TP photodetector shows a pronounced dip to *R* ≈ 56.6% at the TP eigenmode of 2.38 eV, with a quality factor of *Q* ≈ 52.7. Unlike the broad PL emission of the reference device, the TP photodetector displays sharp, spectrally narrow emission. c) Emission decay curves of the reference device (red squares) and the TP photodetector (green squares) measured at the photon energy of 2.38 eV. The curves are fitted with the one‐term exponential decay model, yielding decay times of τ_*ref*_ = 1.38 ns for the reference device and τ_*TP*_ = 0.43 ns for the TP photodetector. The TP structure remarkably shortens the photon lifetime at the resonant energy of the TP eigenmode, indicating an enhanced emission rate and higher absorption probability compared to the reference device.

Since the device's performance is directly determined by the photon‐exciton coupling effect, which is closely tied to the optical quality of the TP structure, we have conducted momentum‐ and time‐resolved photoluminescence (PL) measurements to assess it. **Figure** **3**a presents the energy‐momentum (i.e., dispersion) relationship of the PL emission (right panel) alongside the corresponding momentum‐resolved reflectivity (left panel) for both the reference device (top panels) and the TP photodetector (bottom panels), as measured using a Fourier spectroscopy setup (see the Experimental Section). In the reference device, a bright excitonic state with a stripe pattern is observed at a PL emission energy of ≈ 2.38 eV. This pattern remains constant across different momenta, reflecting the omnidirectional reflection characteristic of the Ag mirror. In contrast, the TP photodetector exhibits a distinct single eigenmode in its reflectivity spectrum, which corresponds to a bending PL emission pattern that shifts with increasing momentum, as described quantitatively in Equation (1). Figure 3b compares the PL and reflectivity spectra of both devices at *k*_||_ = 0. While the quality factor of the TP photodetector, estimated from its reflectivity profile, is relatively low (*Q* ≈ 52.7), the precise spectral alignment between the TP eigenmode and the emission energy of the CsPbBr_3_ QDs significantly reduces the photonic mode volume.^[^ ^52^ ^]^ This reduction strengthens the light‐matter coupling, as evidenced by the sharper emission with a much narrower spectral bandwidth (FWHM ≈ 40 meV) compared to the broader bandwidth of the reference device (FWHM ≈ 110 meV). Moreover, the decay time of the TP photodetector is substantially shorter, decreasing from τ_*ref*_ = 1.38 ns in the reference device to τ_*TP*_ = 0.43 ns (Figure 3c). Using these decay times, the Purcell factor (*F_P_*), defined as *F_P_* = τ_*ref*_/τ_*TP*_,^[^ ^53^ ^]^ is calculated to be *F_P_* = 3.21. This enhanced Purcell factor indicates a significant increase in the spontaneous emission rate in the TP device, driven by its on‐resonance oscillation. The higher emission rate also suggests an increased absorption probability in the CsPbBr_3_ QDs absorber, as predicted by Einstein's coefficient,^[^ ^54^ ^]^ which link radiative emission and optical absorption. These findings are consistent with the principle that higher emission rates correlate with higher absorption probabilities, ultimately leading to improved photodetection performance in the TP photodetector.

**Figure 4:**
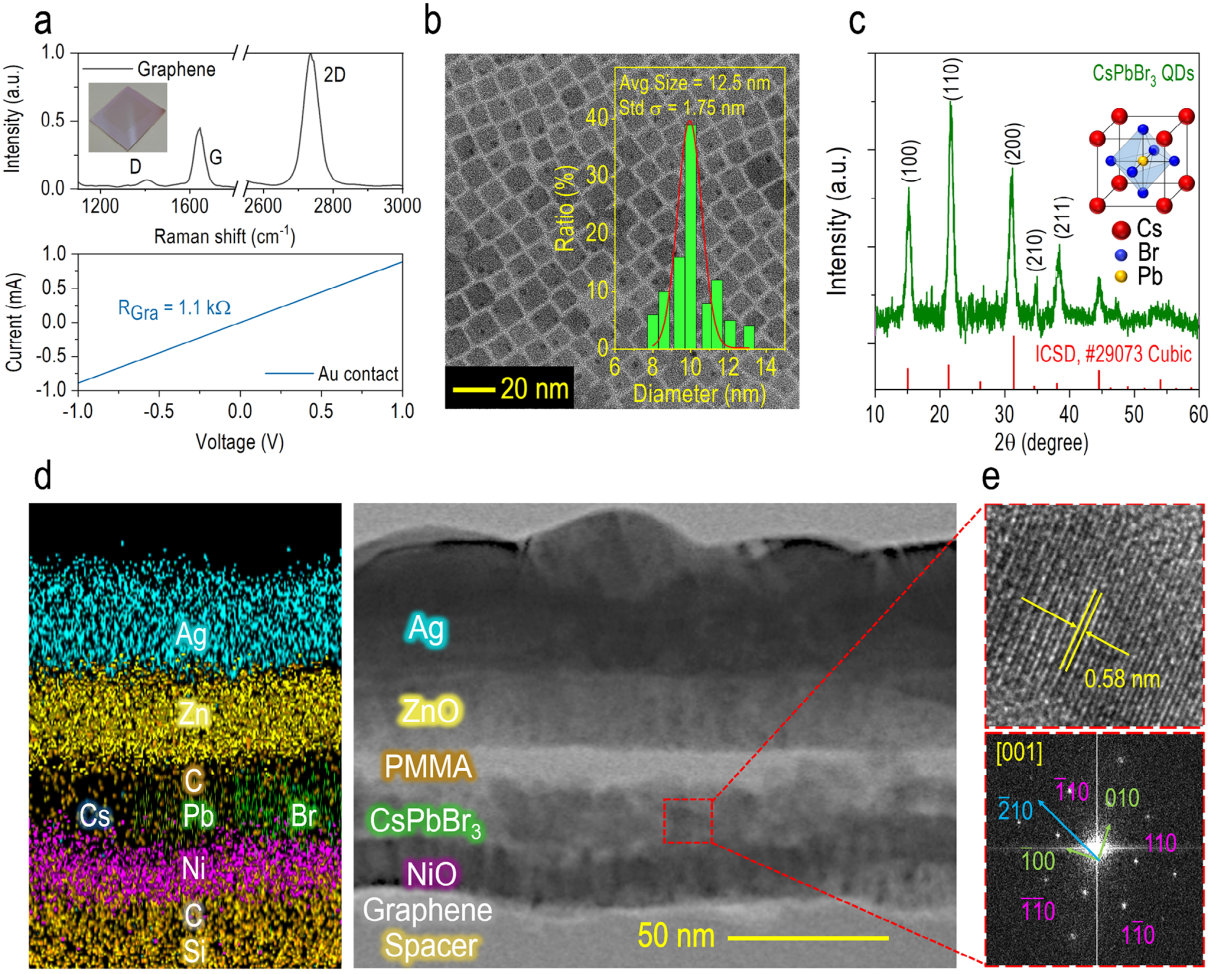
CsPbBr3 QD‐based TP photodetector structure properties. a) Raman spectrum (top panel) and *I–V* characteristics (bottom panel) of SLG transferred onto the DBR. The inset displays a photograph of the actual sample after the transfer process. The *I–V* characteristics were measured by probing the Au pads deposited on the SLG. b) TEM image of the CsPbBr_3_ QDs dispersed onto a carbon‐coated copper grid. A static size distribution analysis of the as‐synthesized QDs reveals an average size of µ = 12.50 nm with a standard deviation of σ = 1.75 nm (inset). c) XRD pattern of the CsPbBr_3_ QDs, matching well with the cubic phase structure (ICSD No. 29073). The inset depicts the 3D crystal structure of cubic CsPbBr_3_, illustrating the orientated atomic lattice arrangement. d) Cross‐sectional TEM image of the TP photodetector (right panel), exposing the region from the SiO_2_ spacer layer to the top Ag mirror, along with the corresponding EDS mapping profile to identify the atomic composition of the device (left panel). e) High‐resolution TEM (HRTEM) image of the CsPbBr_3_ QDs (top panel) and the corresponding fast Fourier transform (FFT) pattern of the basal (001) facet (bottom panel).

Prior to evaluating the photoresponse performance of the CsPbBr_3_ QD‐based TP photodetector, we first examined the structural properties of its primary components. **Figure** **4**a presents the Raman spectrum (top panel) and the *I–V* characteristic (bottom panel) of the SLG transferred onto the DBR surface. The inset of Figure 4a shows a photograph of the SLG on the DBR, where a distinct colored boundary outlines the SLG area, confirming the successful transfer process. Au electrodes were subsequently deposited on the SLG to establish Ohmic contacts. The Raman spectrum exhibits the characteristic graphene peaks, including the D, G, and 2D bands, located at 1402.5,  1645.7, and 2735.8 cm^−1^, respectively. The intensity ratio of the 2D to G bands (*I*_2*D*_/*I_G_* = 2.23) verifies the monolayer nature of the transferred graphene film, while the D to G band intensity ratio (*I_D_*/*I_G_* = 0.15) suggests high‐quality feature with moderate intrinsic defects, as it inversely correlates with defect concentration and grain size.^[^ ^55^ ^]^ This assessment is further supported by the linear *I–V* curve measured in the SLG, which has an acceptable resistance of *R_SLG_* = 1.1 kΩ. The successful transfer of SLG is important, as our simulations indicate that the physical thickness between the Ag and DBR reflectors must be kept below 70 nm to efficiently excite the TP eigenmode. Conventional transparent conducting layers, such as indium tin oxide (ITO) or fluorine‐doped tin oxide (FTO), are too thick and therefore unsuitable for this study. The morphology and homogeneity of the synthesized CsPbBr_3_ QDs were characterized using transmission electron microscopy (TEM). For the analysis, the CsPbBr_3_ QDs were dissolved in toluene and dispersed onto a carbon‐coated copper grid for imaging (Figure 4b). TEM analysis reveals that the CsPbBr_3_ QDs are highly monodisperse, with a monoclinic crystal structure, and an average size of µ = 12.50 nm (standard deviation σ = 1.75 nm, inset of Figure 4b). Given the exciton Bohr radius of CsPbBr_3_ perovskite is around 7 nm,^[^ ^56^ ^]^ strong quantum confinement effects are expected (see Figure S6, Supporting Information), beneficial to the enhanced photon–exciton coupling. The crystallinity of the CsPbBr_3_ QDs was further confirmed by X‐ray diffraction (XRD) analysis (Figure 4c), which matches the cubic perovskite structure belonging to the $Pm\bar{3}m\left(221\right)$ space group (ICSD No. 29073) without any detectable secondary phases. The inset provides a detailed schematic of the cubic phase structure, illustrating the 3D framework of corner‐sharing octahedra with Cs^+^ ions occupying the cuboctahedral cavities. Figure 4d shows the cross‐sectional TEM image of the TP photodetector (right panel). The inspection sample was prepared using focused‐ion beam (FIB) milling, exposing the region from the SiO_2_ spacer layer to the top Ag mirror. The clean interfaces with distinct contrasts between adjacent layers confirm the successful fabrication of the photodetector, achieved through a series of processes including sputtering, spin‐coating, and transfer techniques. Energy‐dispersive X‐ray spectroscopy (EDS) mapping further verifies the atomic composition of the device (left panel, Figure 4d). Expected signals corresponding to the respective atomic elements with spatially uniform distributions were detected throughout the structure, including Cs, Pb, and Br signals appearing in the CsPbBr_3_ QDs absorber, validating the accurate assembly of the TP photodetector. High‐resolution TEM (HRTEM) image (top panel, Figure 4e), along with its corresponding fast Fourier transform (FFT) pattern of the basal (001) facet (bottom panel, Figure 4e), provide additional evidence of the highly crystalline cubic phase of the CsPbBr_3_ QDs. The measured interplanar spacing of 5.8 Å corresponds to the (200) plane, consistent with the XRD results shown in Figure 4c. Collectively, these results demonstrate the high crystallographic quality of the CsPbBr_3_ QDs and their successful integration into the TP photodetector.

### 2.3 Photoresponse Performance and Color Selectivity of the CsPbBr_3_ QD‐Based TP Photodetector

**Figure 5:**
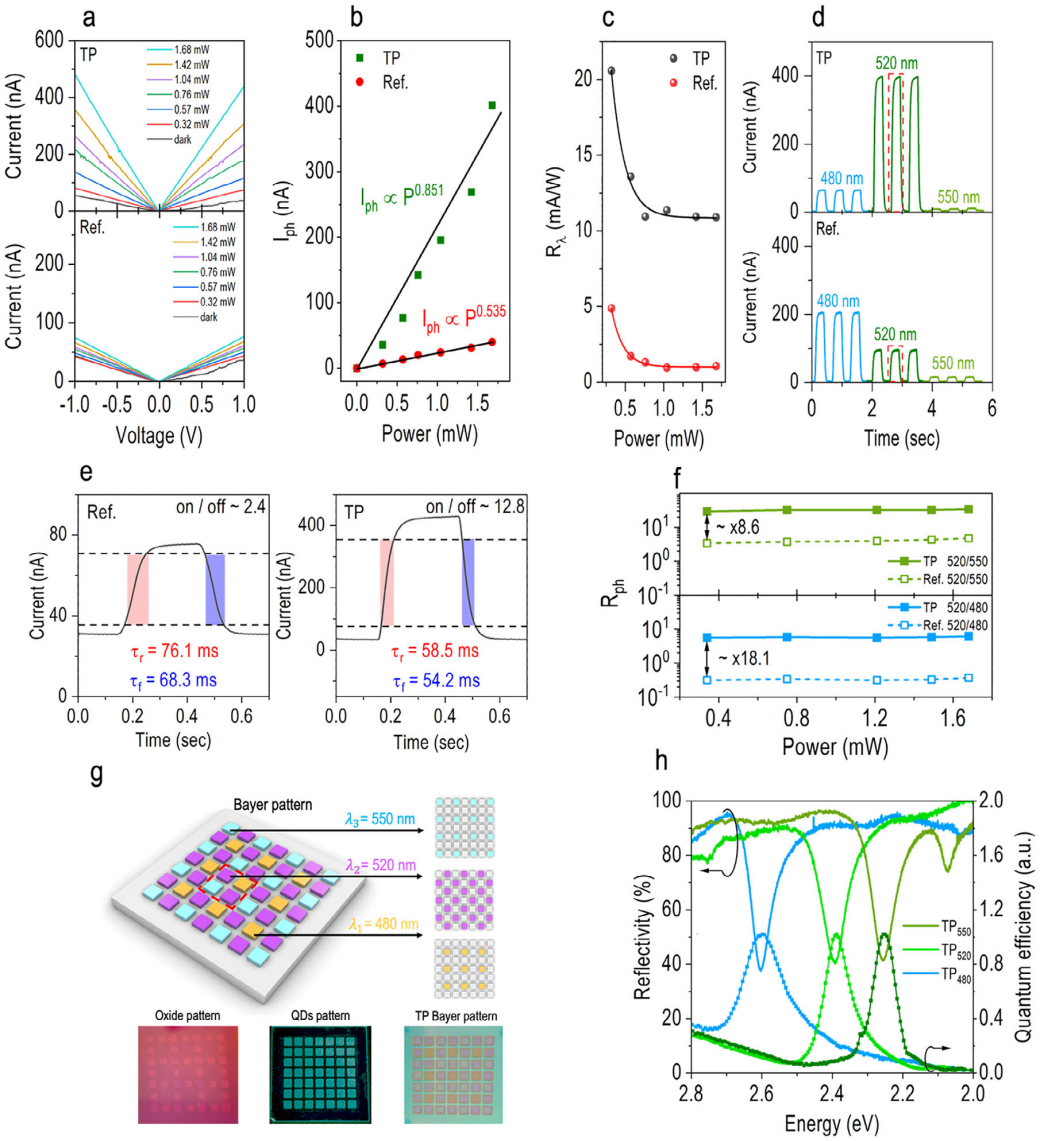
Photoresponse performance and Bayer filter pattern of CsPbBr_3_ QD‐based TP photodetector. a) *I–V* characteristics for the reference device (bottom panel) and the TP photodetector (top panel) under green‐light illumination (λ  = 520 nm) with varying optical power (0.32–1.68 mW). b) Power‐dependent photocurrent extracted at + 1 V for both devices, following a power‐law relationship, $I_{ph}∼P^{\alpha }$. c) Responsivity (*R*_λ_) as functions of incident power for the reference device (red dots) and TP photodetector (black dots), measured at a forward bias of + 1 V. d) Temporal photoresponse to pulsed illumination (power: 1.68 mW, pulse width: 300 ms, duty cycle: 50%) at different excitation wavelengths (λ  =  480/520/550 nm), comparing the performance between the reference (bottom panel) and TP devices (top panel). e) Enlarged view of photoresponse profiles under on‐resonance illumination (λ  = 520 nm), highlighting the rise time (τ_*r*_), fall time (τ_*f*_), and on/off photocurrent ratio for both devices. f) Photocurrent ratio (*R_ph_*) for on‐resonance illuminations (λ  = 520 nm) compared to off‐resonance illuminations (λ  =  550 nm, top panel; λ  = 480 nm, bottom panel) as a function of incident power for both devices. g) TP photodetectors with three distinct resonant eigenmodes properly arranged to form the Bayer filter pattern. The photographs below show each fabrication step involved in creating the Bayer pattern. Each TP device pixel features a lateral dimension of 1.7 mm ×  1.7 mm, with an inter‐pixel spacing of 0.6 mm.This configuration yields a center‐to‐center pixel pitch of 2.3 mm, corresponding to an estimated spatial resolution of ≈ 11.5 pixels per inch (PPI). h) Reflectivity and quantum efficiency for each pixel in the Bayer filter pattern, with three distinct reflective dips observed at 2.58 eV (λ  =  480 nm), 2.38 eV (λ  =  520 nm), and 2.25 eV (λ  =  550 nm), perfectly aligning with the corresponding quantum efficiency peaks.

**Figure** **5**a presents the variations in the *I–V* characteristics of the reference device (bottom panel) and TP photodetector (top panel) under green‐light illumination (λ  =  520 nm) with optical power (*P*) ranging from 0.32 to 1.68 mW. Both devices exhibit a progressive increase in photocurrent in response to the incremental optical power, which is attributed to the generation of more electron‐hole pairs in the CsPbBr_3_ QDs at higher incident powers. The *I–V* characteristics of both the reference and TP photodetector devices exhibit distinct rectifying behavior, suggestive of a well‐formed p–n junction within the device structure (see Figure S7, Supporting Information). Notably, the TP photodetector demonstrates an enhanced performance over the reference device, with its photocurrent at +1 V rising by two orders of magnitude under illumination relative to the dark condition, underlining its superior photoresponse capability despite utilizing an ultrathin CsPbBr_3_ QD absorber only two monolayers thick. Figure 5b further depicts the power‐dependent photocurrent extracted at +1 V for both devices, which follows a power‐law relationship $I_{ph}∼P^{\alpha }$. Here, the photocurrent *I_ph_* is defined as *I_ph_* =  *I_iillumination_*  −  *I_dark_*, representing the difference between current under illumination and in the dark. The power‐law exponent α is determined to be ≈ 0.535 for the reference device and ≈ 0.851 for the TP photodetector. This higher α value for the TP photodetector reflects enhanced optical absorption due to strong photon–exciton coupling, leading to improved linearity of its photocurrent response and greater optoelectronic efficiency at elevated incident power. Figure 5c provides a detailed analysis of the responsivity (*R*_λ_) characteristics for both devices (see Note S1, Supporting Information). At all power levels, the TP photodetector consistently demonstrates higher *R*_λ_ than the reference device. The observed decrease in *R*_λ_ with increasing *P* is attributed to the saturated absorption of the CsPbBr_3_ QDs at higher power levels. Consequently, under the lowest power illumination (*P*  =  0.32 mW), the maximum responsivity values recorded were *R*_λ_ = 4.87 and 20.61 mA W^−1^ for the reference and the TP photodetector devices, respectively.

To validate the enhanced color selectivity of the TP photodetector, we evaluated and compared photocurrent variations of both devices under light pulses with on‐resonance (λ  =  520 nm) and off‐resonance (λ  =  480/550 nm) wavelengths, as shown in Figure 5d. When illuminated at the on‐resonance wavelength, the TP photodetector generates a higher photocurrent compared to off‐resonance illuminations (top plot, Figure 5d). This indicates that light oscillating at the resonant frequency of the TP eigenmode couples more effectively into the embedded CsPbBr_3_ QDs, leading to greater absorption and yielding a higher photocurrent. Notably, despite the inherently stronger absorption of CsPbBr_3_ QDs at shorter wavelengths (top plot, Figure 1b), which typically results in higher photocurrent for the reference device under shorter‐wavelength illumination (bottom panel, Figure 5d), the TP photodetector still outperforms under on‐resonance illumination. In fact, it produces a higher photocurrent compared to shorter‐wavelength off‐resonance conditions. Consequently, the TP photodetector demonstrates a superior on/off ratio of ≈12.8 under on‐resonance illumination (right panel, Figure 5e), surpassing the reference device, which achieves an on/off ratio of only ≈2.4 under the same condition (left panel, Figure 5e). Moreover, the rise time (τ_*r*_) and fall time (τ_*f*_), defined as the time required for the photocurrent to transition between 10% and 90% of its maximum value, are considerably faster in the TP photodetector than in reference device. For the TP photodetector, τ_*r*_ =  58.5 ms and τ_*f*_ = 54.2 ms, while for the reference device, τ_*r*_ = 76.1 ms and τ_*f*_ = 68.3 ms. This observation suggests that the carrier dynamics of the TP photodetector are shaped by the interaction between incident photons and perovskite excitons, resulting in a shorter response time for generating or decaying photocurrent. Despite of that, a performance disparity remains when compared to commercially available Si‐based photodetectors, which may stem from the high defect density inherently in the CsPbBr_3_ QDs (≈10^15^–10^18^ cm^−3^).^[^ ^57^ ^]^ These defects act as recombination centers that trap photogenerated carriers, impede carrier mobility, and result in increased dark currents and sluggish photoresponse dynamics. Defect‐mitigation strategy, such as surface ligand exchange treatments^[^ ^58^, ^59^ ^]^ or the growth of single‐crystalline perovskite films via chemical vapor deposition,^[^ ^60^, ^61^ ^]^ would be a possible way to narrow the performance gap. Figure 5f shows the photocurrent ratio (*R_ph_*) measured under on‐resonance illumination (λ  =  520 nm) compared to off‐resonance illuminations at λ  =  550 nm (top panel) and λ  =  480 nm (bottom panel). In both cases, *R_ph_* is plotted as a function of incident power for the reference device (open squares) and TP photodetector (solid squares). Under on‐resonance (λ  =  520 nm) and off‐resonance (λ  =  550 nm) conditions (top panel, Figure 5f), the reference device achieves an *R_ph_* of ≈3.39, while the TP photodetector achieves a much higher *R_ph_* of ≈29.37 across all power levels. This corresponds to an ≈8.6‐fold enhancement in photoresponse at the on‐resonance wavelength, entirely attributed to the employment of the TP structure. The enhancement becomes even more pronounced when comparing on‐resonance illumination at λ  =  520 nm to off‐resonance illumination at λ  =  480 nm (bottom panel, Figure 5f), where the *R_ph_* enhancement reaches ≈18.1‐fold. This dramatic increase arises because the reference device generates more photocurrent at shorter wavelengths due to the stronger intrinsic absorption of CsPbBr_3_ QDs, resulting in a lower *R_ph_* (≈0.31) for the reference device. Similarly, the *R_ph_* of the TP photodetector also decreases to ≈5.63 under these conditions, consistent with the observations in Figure 5d. These findings collectively demonstrate the TP photodetector's impressive ability to swiftly and efficiently convert absorbed energy into photocurrent. More importantly, they confirm its exceptional capability in achieving high photoresponse at the target wavelength without the need for traditional color filters. A systematic comparison of key photoresponse performance metrics, including responsivity, detectivity, and photocurrent selectivity, between the proposed TP photodetector and previously reported perovskite QD‐based photodetectors is summarized in Table S1 (Supporting Information). Beyond demonstrating competitive responsivity and detectivity, our TP device offers substantially improved wavelength selectivity, making it promising for high‐performance, narrowband photodetection applications.

To demonstrate the potential of our TP photodetector as a practical image sensor, we designed and fabricated devices optimized for three distinct eigenmodes corresponding to optical wavelengths of λ  =  480, 520, and 550 nm. The extension of spectral response into the red region was constrained by the intrinsic absorption characteristics of the CsPbBr_3_ QDs. These photodetectors were arranged in a sequential mosaic pattern inspired by the Bayer filter configuration, which strategically incorporates a higher proportion of green filters relative to red and blue to enhance luminance resolution and align with the human eye's increased sensitivity to green light.^[^ ^62^ ^]^ In order to replicate the perceptual weighting embedded within the Bayer pattern, the TP photodetector array was intentionally engineered to exhibit augmented responsivity at λ  =  520 nm. This spectral emphasis not only reflects the spectral sensitivity profile of the human visual system but also stresses the wavelength‐selective tunability enabled by the proposed TP photodetector array. As shown in Figure 5g, each λ  =  550 nm eigenmode is surrounded by four λ  =  520 nm eigenmodes and four λ  =  480 nm eigenmodes. Similarly, each λ  =  480 nm eigenmode is bordered by four λ  =  550 nm eigenmodes and four λ  =  520 nm eigenmodes. For λ  =  520 nm eigenmodes, each is flanked by two λ  =  550 nm, four λ  =  520 nm eigenmodes, and two λ  =  480 nm eigenmodes. The basic repeating unit cell, as marked in Figure 5g, consists of one λ  =  480 nm eigenmode and one λ  =  550 nm eigenmode arranged along one diagonal, with two λ  =  520 nm eigenmodes positioned along the other diagonal. To fabricate the TP photodetector array with a Bayer filter pattern, we carefully control the thickness of the bottom DBR spacer (*d_spacer_*) assigned to each pixel within the unit cell. This ensured that each pixel was precisely tuned to excite the desired TP eigenmode at the target wavelength. As the bottom of Figure 5g, the fabrication process is documented step by step with photographs, including (1) the deposition of the SiO_2_ spacer layer with the specified thickness for each pixel (lower left), (2) the subsequent patterning of the CsPbBr₃ QD absorber (lower center), and (3) the deposition of the Ag mirror to finalize the Bayer filter pattern (lower right). Notably, the differences between the pixels in the Bayer filter pattern remain visually indistinguishable until the Ag mirror layer is fully deposited. Figure 5h presents the reflectivity and quantum efficiency for each pixel in the Bayer filter pattern. Three distinct reflective dips, occurring at 2.58 eV (λ  =  480 nm), 2.38 eV (λ  =  520 nm), and 2.25 eV (λ  =  550 nm), align perfectly with the peak quantum efficiency. The measured quantum efficiency of the TP device generally falls within the range from ≈ 0.59% to 2.37%. This performance, while moderate, indicates significant room for enhancement through further optimization using surface treatment or passivation strategies.^[^ ^58^, ^59^ ^]^ These results demonstrate that our TP photodetector achieves high wavelength selectivity while maintaining excellent adaptability for multipixel integration, making it a promising candidate for practical image sensor applications. Moreover, the selectively enhanced responsivity at specific photon energies endows the device with significant potential for neuromorphic vision systems, where color‐discriminative learning and pattern recognition are required. Such functionalities remain challenging to achieve using conventional photodetector technologies, which generally lack both spectral selectivity and the capability to emulate synaptic plasticity.

### 2.4 Photonic Neural Synapse and Facial Recognition Using CsPbBr_3_ QD‐Based TP Photodetector

**Figure 6:**
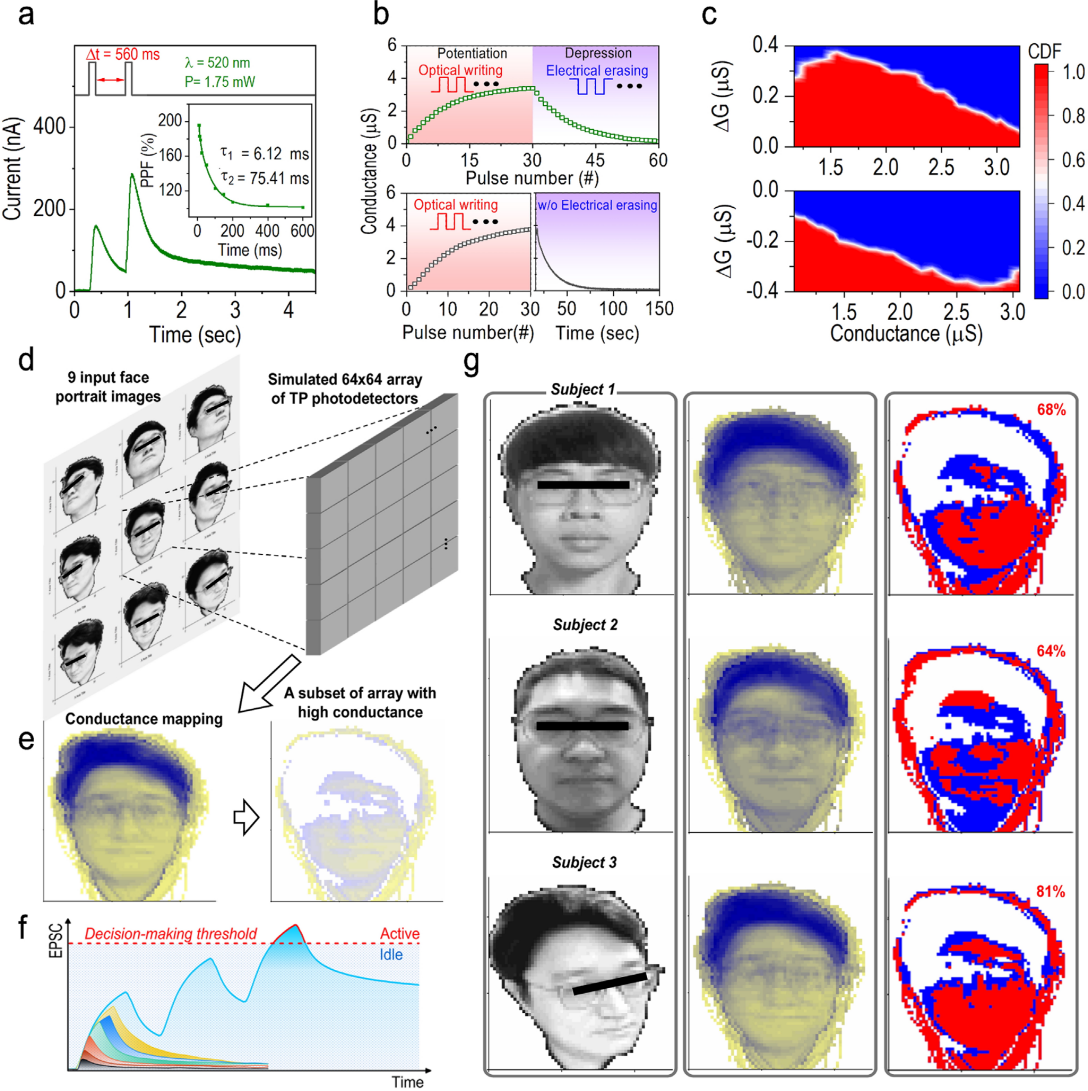
Synaptic dynamics and facial recognition using CsPbBr_3_ QD‐based TP photodetector. a) EPSC response induced by two successive light pulses (λ  = 520 nm, *P* = 1.75 mW, *t_p_* = 100 ms, and Δ*t* = 560 ms) in the TP photodetector. The inset illustrates the variation of the PPF index as a function of *∆t*, fitted using a double exponential model. The extracted time constants, corresponding to the rapid and slow decay phases, are τ_1_ = 6.12 ms and τ_2_ = 75.41 ms, respectively. b) LTP and LTD processes triggered by applying 30 consecutive optical and electrical pulses to the TP photodetector (top panel). The optical pulses are identical to those used in (Figure a), whereas each electrical pulse is applied with a bias of −40 mV and consists of on and off durations of 10 ms each. Natural decay behavior of the potentiated conductance state in the absence of electrical erasure (bottom panel). c) The CDF plot of ∆*G* versus *G* for the TP photodetector, illustrating the probability of a specific ∆*G* value at a given *G* during the LTP (top) and LTD (bottom) processes. d) Illustration of the training phase for facial recognition, where optical signals from nine grayscale images of the same face, captured from different angles, were input to a simulated 64 × 64 array of TP photodetectors. e) Reconstructed mapping image of synaptic weights in correspondence to the nine images after the training phase (left panel). A subset of the array was selected to minimize redundant information and enhance the facial recognition process (right panel). f) Decision‐making threshold setting in the LTP process of the TP photodetector to differentiate active and idle status in the simulated model. g) Demonstration of facial recognition tests using three input faces (left column) processed through a trained threshold model, and the corresponding conductance mappings were obtained (middle column). TP photodetector array exhibits a high activation rate when tested with the target face of Subject 3, while showing relatively low activation rates for the other test images of Subjects 1 and 2 (right column).

Referring to the energy diagram of the TP photodetector in Figure 1d, the PMMA layer, situated between the ZnO electron transport layer and the CsPbBr_3_ QDs absorber, introduces a barrier height in the conduction band. This *E_C_* discontinuity traps and accumulates photogenerated electrons, thereby effectively regulating the current flow to enable synaptic functionalities. In this study, we exploit this feature to emulate synaptic dynamics and perform facial recognition tasks. To demonstrate the potential of the TP photodetector as a fundamental artificial synapse, we systematically investigate its synaptic plasticity, including several key characteristics such as EPSC, PPF, and LTP and LTD processes. **Figure** **6**a depicts the PPF dynamic of the TP photodetector, a form of short‐term plasticity (STP) induced by two closely spaced light pulses with a temporal interval (Δ*t*) of 560 ms. In this experiment, the device is stimulated by light pulses with a wavelength of 520 nm (power: *P*  =  1.75 mW, pulse width: *t_p_* = 100 ms). Accordingly, the peak EPSC generated by the second pulse (*A*_2_) exceeds that of the first (*A*_1_). This enhancement arises from residual photogenerated carriers from the first pulse, which, before stabilizing through recombination or diffusion process, combine with carriers from the second pulse to amplify the EPSC and synaptic weight. The PPF index, defined as PPF  =  (*A*_1_/*A*_2_)) × 100%, is plotted as a function of Δ*t* in the inset of Figure 6a. As Δ*t* increases, the PPF index decreases, following a decay trend similar to that observed in biological synapses. The decay behavior is modeled using a double exponential function^[^ ^63^ ^]^:

(2) $$PPF\;index=C_{0}+C_{1}\;exp\left(-\Delta t/\tau_{1}\right)+C_{2}\;exp\left(-\Delta t/\tau_{2}\right)$$

where *C*_0_, *C*_1_, and *C*_2_ represent the initial facilitation magnitudes, while τ_1_ and τ_2_ denote the relaxation times for the rapid and slow decay phases, respectively. The extracted values, τ_1_ = 6.12 ms and τ_2_ = 75.41 ms, reveal that the fast decay phase is an order of magnitude quicker than the slow phase, a phenomenon that resembles the dynamics of biological synaptic decay. The TP photodetector also demonstrates robust synaptic plasticity under sequential optical and electrical pulse stimuli (top panel, Figure 6b). Potentiation occurs when 30 consecutive light pulses, each having the identical condition as used in Figure 6a, are applied. In contrast, depression is induced by 30 consecutive electrical pulses at −40 mV, with on and off times of 10 ms each. This behavior arises from the gradual relaxation of the potentiated conductance, which returns to its initiate state over ≈75 s (bottom panel, Figure 6b). The negative electrical pulses are hence essential in suppressing the potentiated state by repelling accumulated electrons, underlining the device's inherent nonvolatile synaptic plasticity in photocurrents. During illuminations, the conductance of the TP photodetector progressively increases with successive light pulses, achieving the LTP. When the light pulses cease and 30 consecutive depression electrical pulses are applied, the conductance decreases gradually, simulating the LTD. The conductance state of both LTP and LTD processes exhibits a nonlinear phenomenon, rapidly increasing during the first 15 input pulses and then approaching saturation with additional pulses. The nonlinearity (β) and the number of effective conductance states (*N_eff_*) of the TP photodetector are quantitatively estimated (see Figure S8, Supporting Information). Here shall be addressed that while the synaptic behavior is fundamentally enabled by the PMMA interlayer, its manifestation can be substantially amplified under on‐resonance illumination onto the TP device. Comparative analysis of synaptic response under resonant and off‐resonant excitation reveals that on‐resonance illumination yields improved synaptic characteristics, as evidenced by a reduced β value and an elevated *N_eff_*. These parameters play a pivotal role in broadening the modulation range of synaptic weight, which is essential for achieving high‐accuracy image recognition in neuromorphic vision applications (see Figure S9, supporting information). Figure 6c presents the cumulative distribution function (CDF) of conductance change (∆*G*) versus conductance (*G*), illustrating the probability of a specific ∆*G* value at a given *G* during the LTP (top panel) and LTD (bottom panel) processes. These plots are derived from conductance updates measured over 10 LTP/LTD cycles (see Figure 6b). During the LTP process, the ∆*G* corresponding to a CDF value of 0.5 (white region) initially increases with *G*, reaching a peak before gradually declining. The region suitable for linear synaptic weight modulation is defined as ∆*G* > 0.1 µS, which corresponds to a conductance range of up to 2.9 µS. This region is achieved within the first 15 light pulses, representing 50% of the effective conductance states (15 out of 30 light pulses). Similarly, during the LTD process, ∆*G* values less than − 0.1 µS are suitable for linear synaptic weight modulation, corresponding to a conductance range of 1.05 − 3.10 µS, also encompassing 50% of the effective conductance states through electrical pulses.

Based on the experimental observation of synaptic dynamics, we further simulated facial recognition capability using a 64 × 64 array of TP photodetectors under the ANN‐based weight‐update expectation thresholding model (see Note S2, Supporting Information).^[^ ^64^ ^]^ This simulation includes both model training and testing phases. During the training phase, nine grayscale portraits of the same face, taken from different angles, were used to train the TP photodetector array (Figure 6d). Each photodetector processed a single pixel from the input optical image, converting it into a presynaptic spike. The plasticity behavior of the post‐synaptic signal across the array was recorded, constructing a conductance mapping learned from the nine images. Synaptic weights at each pixel were updated and optimized to extract distinct facial features from various orientations (left panel, Figure 6e). To enhance facial recognition and reduce the influence of redundant information, a subset of the array with high conductance values was selected to emphasize key facial outlines (right panel, Figure 6e). This selection aligned with the transient potentiation behaviors of the TP photodetector, enabling a decision‐making threshold to be applied at each pixel (Figure 6f). When the EPSC generated by consecutive light pulses exceeded this threshold, the pixel was marked as active status, indicating recognition of the visual signal. Otherwise, the pixel was labeled as idle. Facial recognition tests were then conducted using this threshold model with three example faces (Figure 6g). When images of the three faces (left column) were fed into the trained threshold model, the corresponding conductance mappings were obtained (middle column). By applying the same threshold level to activate outlined features, the activation rate between the test and target images was calculated (right column). For images of other subjects, the activation rates were 68% and 64% for subjects 1 and 2, respectively, indicating limited similarity to the target. In contrast, the activation rate exceeded 80% when testing with the target face of subject 3. This difference in activation rates between test and target images can be further enhanced by increasing the number of test images used to train the threshold model, which amplifies the contrast of the target's facial outlines. These results demonstrate that our TP photodetector array effectively learns essential visual features for recognizing target images, even with variations in facial orientation.

## 3 Conclusion

In conclusion, we have demonstrated that the photodetection and color selectivity of the CsPbBr_3_ QD‐based photodetector can be significantly enhanced by monolithically integrating a monolayer‐scale QD absorber into a TP structure. Under on‐resonance illumination, strong photon–exciton coupling was observed, evidencing that the enhanced Purcell factor associated with spontaneous emission increases the absorption probability, as predicted by Einstein's coefficients. The resonant energy of the TP eigenmode can be precisely tuned within the DBR's photonic stopband, enabling highly selective responses to specific incident wavelengths. To further enhance functionality, we arrange the TP photodetector array as a Bayer filter pattern, where three distinct reflective dips align perfectly with the peak quantum efficiency at each corresponding photosite, making it highly promising for wavelength division multiplexing (WDM) and multipixel integration systems.^[^ ^65^, ^66^ ^]^ More interestingly, introducing a PMMA layer in the TP photodetector creates a conduction band discontinuity, forming a barrier height that allows photogenerated electrons to accumulate under consecutive light pulse stimulations. This enables multilevel conductance modulation and efficient current regulation. We also simulate image recognition functionality using the TP photodetector array as synaptic devices with an ANN‐based weight‐update expectation thresholding model. These results represent a significant advancement in leveraging all‐inorganic perovskite materials for compact, color‐filter‐free image sensors, and establish a foundation for integrating photonic neural computation on a single platform.

## 4 Experimental Section

### Synthesis of CsPbBr_3_ QDs

In this study, CsPbBr_3_ QDs were synthesized using the hot injection method. To prepare the Cs‐oleate solution, 407 mg of cesium carbonate (Cs_2_CO_3_), 20 mL of octadecene (ODE), and 2 mL of oleic acid (OA) were combined in a three‐neck flask. The mixture was degassed at 120 °C for 1 h and then heated to 150 °C under a nitrogen atmosphere for 3 h to ensure complete dissolution of Cs_2_CO_3_ in the ODE, forming the Cs‐oleate precursor solution. Simultaneously, 400 mg of lead bromide (PbBr_2_), 1400 mg of zinc bromide (ZnBr_2_), 7 mL of OA, 7 mL of oleylamine (OLA), and 20 mL of ODE were placed in another three‐neck flask and dried under nitrogen at 120 °C for 1 h. Once the PbBr_2_ was fully dissolved at 180 °C, 3 mL of the Cs‐oleate precursor solution was quickly injected into the flask, initiating the reaction. The reaction was allowed to proceed for 30 s before the flask was rapidly cooled in an ice bath. The resulting precipitate was collected by centrifugation and redispersed in toluene, yielding the CsPbBr_3_ QDs solution.

### Single Monolayer Graphene Transfer Using Wet Chemical Processes

The monolayer graphene, grown on Cu foil via chemical vapor deposition (CVD), was purchased from Graphene Supermarket (single‐layer graphene on copper foil: 4 in. × 4 inn.). After cutting it to the desired size, a layer of PMMA was spin‐coated onto the graphene surface to serve as a support, and then the sample was dried in an oven at 50 °C for 20 min. During spin‐coating, residual PMMA often adhered to the backside of the Cu foil, which could affect the uniformity of the subsequent wet etching process. To address this, the PMMA on the backside was removed using oxygen plasma etching at 100 W for 3 min. The Cu foil was then immersed in an etching solution of (NH_4_)_2_S_2_O_8_ (0.37 mol L^−1^) until it was fully etched. The graphene layer, still protected by PMMA, was rinsed in deionized water to remove any residual etching solution. It was then scooped out directly onto the target substrate and heated at 100 °C to remove any remaining moisture. Finally, the sample was immersed in acetone to remove the PMMA support layer, leaving behind a clean and transparent graphene conductive film.

### Device Fabrication of TP Photodetector

The DBR grown on a glass substrate was cleaned using ultrasonic treatment in acetone, isopropanol, and deionized water for 10 min each. Subsequently, the SLG was transferred onto the DBR surface through wet chemical processing to serve as the current‐spreading layer. A 20 nm NiO hole transport layer (HTL) was deposited via RF sputtering under a base pressure of 5.0 × 10^−7^ Torr, a working pressure of 6.5 × 10^−3^, and a sputtering power of 100 W. Argon gas was introduced at a flow rate of 30 sccm as the inert sputtering medium. A CsPbBr₃ square array (20 nm thick, 1.7 mm × 1.7 mm) was then fabricated through direct photocatalytic patterning method.^[^ ^67^ ^]^ The patternable ink solution was prepared by mixing a 20 mg mL^−1^ CsPbBr₃ solution with a 0.025 m PTMP solution in toluene in a 9:1 ratio. This mixture was spin‐coated onto the NiO layer at 2000 rpm for 60 s. A shadow mask with a square array pattern was applied, and the sample was exposed to UV light (365 nm, 27 mW cm^−2^) for 30 s. After that, unexposed CsPbBr₃ QDs were developed with toluene, followed by baking on a hot plate at 60 °C for 10 min to form the square array. Next, a 1.5 wt% PMMA electron‐blocking layer was spin‐coated onto the CsPbBr₃ patterns at 5000 rpm for 60 s, followed by the deposition of a 20 nm ZnO electron transport layer (ETL) via RF sputtering. Finally, the anode (Ag, 40 nm) and cathode (Au, 50 nm) were thermally evaporated onto the ZnO layer and the SLG edge, respectively, completing the TP photodetector fabrication. To enhance compatibility with established CMOS fabrication processes, the Ag and Au electrodes employed as n‐ and p‐type contacts here may be substituted with aluminum (Al)^[^ ^68^ ^]^ and titanium nitride (TiN),^[^ ^69^ ^]^ respectively. Al offers comparable optical reflectivity and a similar work function to Ag, thereby maintaining the TP resonance and supporting efficient electron extraction. Meanwhile, TiN, owing to its high work function, represents a suitable alternative to Au, enabling reliable hole collection while remaining CMOS‐compatible.

### Electromagnetic Waves Simulation

In this work, the propagation of electromagnetic waves within the TP photodetector was simulated using COMSOL Multiphysics, a commercially available software based on the finite‐element method. A 2D frequency‐domain electromagnetic model was adopted, with Floquet periodic boundary conditions applied to both sides of the unit cell. Plane waves were incident perpendicularly from the top of the structure. The wavelength‐dependent refractive indices and extinction coefficients of the materials used in the simulation were determined using an ellipsometer (J.A. Woollam Inc., M2000‐DI). To ensure accurate boundary handling, perfectly matched layers were applied at all edges, and the mesh grid was configured with a resolution of 10 nm along the *x*‐axis and 1 nm along the *y*‐axis.

### Measurement Details

The microscale angle‐resolved reflectivity and PL spectra were measured using a home‐built micro‐photoluminescence system, capable of real‐space and Fourier‐space imaging to reveal energy‐momentum dispersion relations. The optical setup employed a 20× objective lens with a numerical aperture (NA) of 0.42, providing an angular range of ±24.8°. For reflectivity measurements, a Xenon white light source (Newport 70515) coupled with a monochromator was focused onto the sample with a beam diameter of ≈10 µm. For PL measurements, the sample was excited by a diode‐pumped, passively Q‐switched laser (Teem Photonics, PNV‐M02510‐1 × 0) with a center wavelength of 355 nm, a pulse duration of 350 ps, and a repetition rate of 1 kHz, generating a focused beam with a diameter of around 5 µm. The reflected and emitted signals were collected through the same objective lens and directed into a spectrometer (Horiba iHR550) with a 550 mm focal length, equipped with an 1800 lines mm^−1^ grating and a liquid‐nitrogen‐cooled CCD camera (256 × 1024 pixels). Steady‐state PL and Raman spectra were also measured using the same spectrometer, with a 355 nm laser for PL excitation and a 532 nm laser for Raman measurements, while the absorption spectrum of the CsPbBr_3_ QDs was recorded using a Newport CS130B‐3‐MC monochromator, equipped with an 819C‐UV‐5.3‐CAL integrating sphere. Time‐resolved photoluminescence (TRPL) measurements were conducted using a time‐correlated single‐photon counting system (PicoHarp 300, PicoQuant) integrated with a spectrometer (iHR320, HORIBA). A 377 nm pulsed laser (P‐C‐375, PicoQuant) operating at an 8 MHz repetition rate and a pump power of 1 µW was used for excitation. The laser beam was focused onto the sample through a 20× objective lens with a NA of 0.42, resulting in a beam spot of ≈5 µm in diameter. The electrical characterization under light illumination was conducted for both the reference device and the TP photodetector using a Keithley 2400 source meter. Light sources included three commercially packaged LEDs with wavelengths of 480, 520, and 550 nm were adopted. Photoresponse measurements were carried out with the assistance of an arbitrary waveform generator (JDS6600‐60 M, Cleqee) and an oscilloscope (Keysight DSOX1204G). To ensure accurate measurement of the incident optical power from the LEDs, an optical power meter (Newport 1919‐R) paired with a photodiode (Newport 818‐UV/DB) was used for verification. To identify the material characteristics, XRD patterns of the perovskite QDs were obtained using a Bruker New D8 Discover multipurpose X‐ray diffraction system, while microstructural images and EDS spectra of the samples were captured with a JEOL JEM‐2100F atomic‐resolution transmission electron microscope operating at 200 kV.

### Statement of Ethics

As confirmed by the Ethics Committee/IRB of National Cheng Kung University, no ethical approval was required for this study. Permission to collect facial images for the purposes of this research was obtained from all participants, who were fully informed about the purposes of the study and the intended use and storage of their images. All participants provided written informed consent for the use and publication of their facial images in this research.

## Conflict of Interest

The authors declare no conflict of interest.

## Supporting information



Supporting Information

## Data Availability

The data that support the findings of this study are available from the corresponding author upon reasonable request.
