# Supplementary material for: Color‐Filter‐Free Image Sensor Using CsPbBr3 Quantum‐Dot‐Based Tamm Plasmon Photodetector for Photonic Synapse Facial Recognition
Source: Adv Sci (Weinh). 2025 Jun 25;12(33):e03464. doi: 10.1002/advs.202503464 (PMC12412571; doi:10.1002/advs.202503464)
Supplement: Supplementary file 1 — Supporting Information [file ADVS-12-e03464-s001.docx]

Supporting Information

Color-filter-free image sensor using CsPbBr_3_ quantum-dot-based Tamm plasmon photodetector for photonic synapse facial recognition

Meng-Cheng Yen, Yung-Chi Yao, Chia-Jung Lee, Shi-Hui Huang, Wei Jie Hong, Yuto Kajino, Hsu-Cheng Hsu, Min-Hsiung Shih, Gong-Ru Lin*, Kaoru Tamada*, Jinn-Kong Sheu*, & Ya-Ju Lee*

M.-C. Yen, Y.-C. Yao, C.-J. Lee, S.-H. Huang, J.-K. Sheu, Y.-J. Lee

Program on Key Materials, Academy of Innovative Semiconductor and Sustainable Manufacturing (AISSM), National Cheng Kung University,

No. 1, University Road, Tainan City 70101, Taiwan
E-mail: [jksheu@ncku.edu.tw](mailto:jksheu@ncku.edu.tw); [yjlee@gs.ncku.edu.tw](mailto:yjlee@gs.ncku.edu.tw)

W.-J. Hong, J.-K. Sheu, Y.-J. Lee

Department of Photonics, National Cheng Kung University,

No. 1, University Road, Tainan City 70101, Taiwan

Y. Kajino, K. Tamada

Institute for Materials Chemistry and Engineering (IMCE), Kyushu University,

744 Motooka, Nishiku, Fukuoka 819-0395, Japan

E-mail: [tamada@ms.ifoc.kyushu-u.ac.jp](mailto:tamada@ms.ifoc.kyushu-u.ac.jp)

M.-H. Shih

Research Center for Applied Sciences, Academia Sinica,

Taipei, 11529 Taiwan

K. Tamada

Advanced Institute for Materials Research (AIMR), Tohoku University,

Sendai 980-8577, Japan

**Supplementary Figure 1**


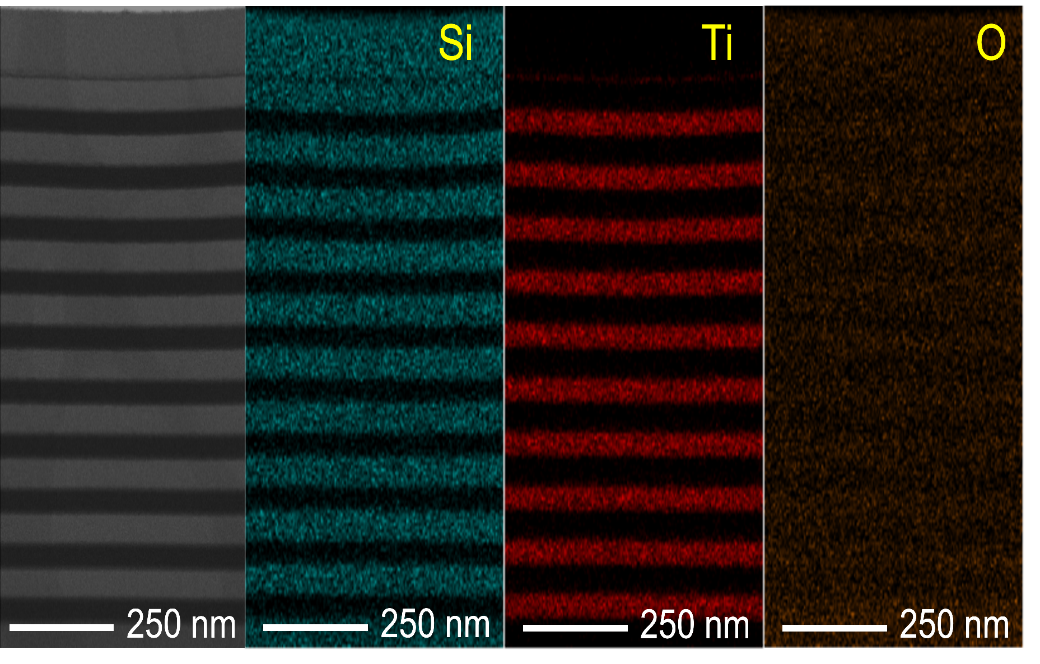


**Figure S1**. The TiO_2_/SiO_2_ DBR structure was analyzed using elemental mapping, revealing an alternating distribution of Ti and Si, while the O element is uniformly distributed throughout the DBR. These findings confirm the precise assembly of the constituent elements in constructing the DBR.

To minimize the optical loss in the resonant cavity and achieve a high-quality TP eigenmode, this work employs a DBR mirror with excellent flatness and high reflectivity. **Figure S1** shows a cross-sectional TEM image of the 10-pair TiO_2_/SiO_2_ DBR dielectric stack, revealing a well-defined periodic multilayered feature. The thicknesses of the functional layers are measured as follows: TiO_2_ (55 nm, dark region) and SiO_2_ (83 nm, bright region). Additionally, EDS elemental mapping (right panel) was conducted on this DBR structure, using Si, Ti, and O as detection signals, confirming the uniform distribution of these elements in accordance with the defined stack.

**Supplementary Figure 2**


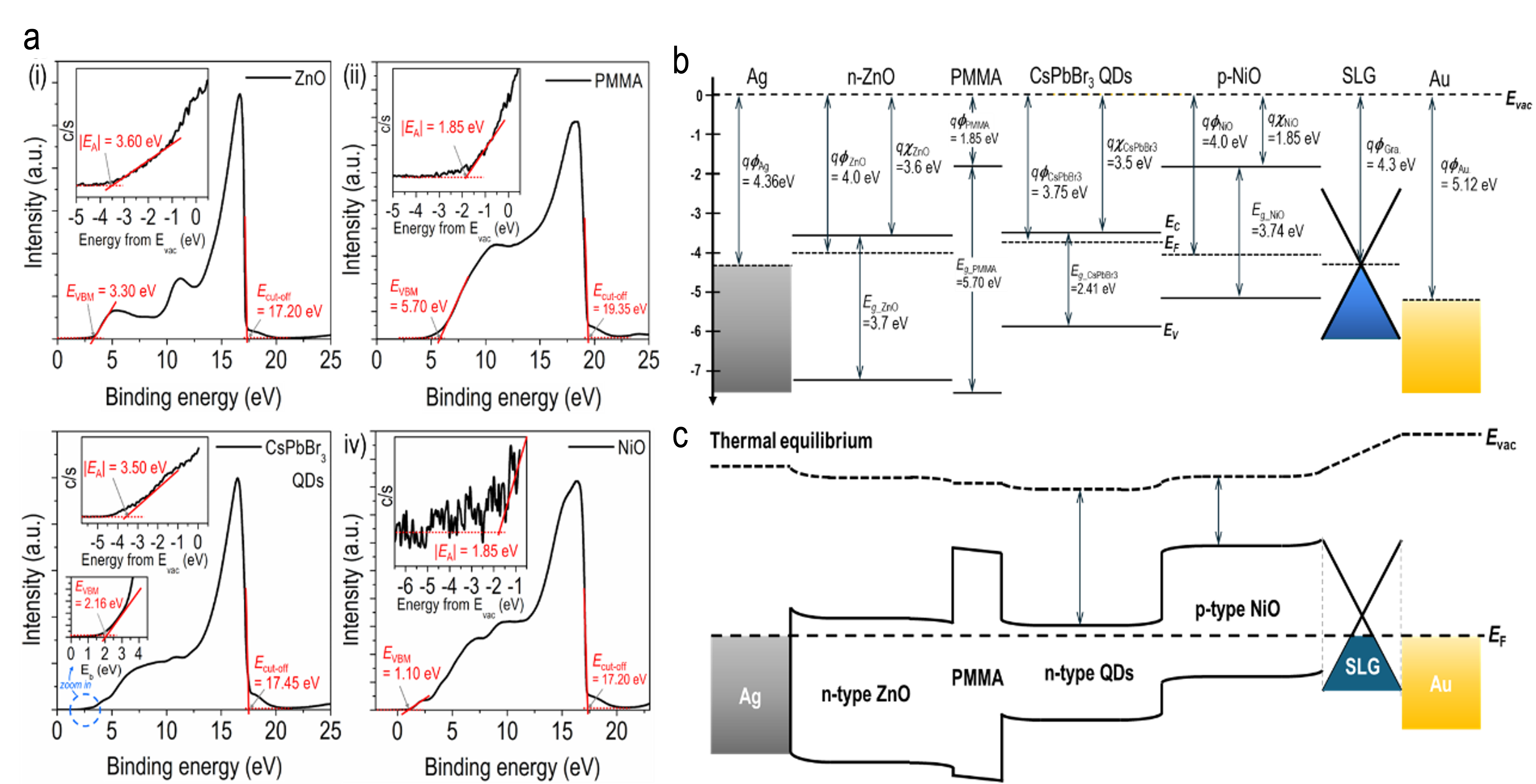


**Figure S2**. **a)** Ultraviolet Photoelectron Spectroscopy (UPS) spectra of (i) ZnO, (ii) PMMA, (iii) CsPbBr_3_ QDs, and (iv) NiO materials. Insets show their corresponding LEIPS spectra. The complete energy-band diagram of the TP photodetector. **b)** before and **c)** after the thermal equilibrium condition.

To elucidate the detailed energy-band diagram of our TP photodetector, we utilized ultraviolet photoelectron spectroscopy (UPS) and low-energy inverse photoemission spectroscopy (LEIPS) to examine the energy band structure of individual constituent components of the device. Specifically, UPS was used to study the occupied electronic states, focusing on the valence band maximum (*E_V_*), and to determine the materials’ work function (*WF*, *qϕ*) and ionization energy (*IE*) of the materials. **Figure S2a** presents the UPS spectra of four materials: (i) ZnO, (ii) PMMA, (iii) CsPbBr_3_ QDs, and (iv) NiO. The work functions of these materials were calculated using **Equations S1** and **S2**:

$IE=h\nu-(E_{cutoff}-E_{VBM})$ (S1)

$WF=IE-E_{VBM}$ (S2)

In these equations, $h\nu$ represents the ultraviolet incident energy (i.e., 21.20 eV). From the UPS spectra in **Figure S2a**, we determined that the cutoff energy (*E_cutoff_* ) and valence band maximum energy (*E_VBM_*) for each material were ZnO: 17.20 eV / 3.30 eV, PMMA: 19.40 eV / 5.70 eV, CsPbBr_3_ QDs: 17.45 eV / 2.16 eV, and NiO: 17.20 eV / 1.10 eV. Using these values in Eqs. (S1) and (S2), the calculated work functions were *WF*_ZnO_ = 4.0 eV, *IE*_PMMA_ = 7.5 eV, *WF*_QD_ = 3.75 eV, and *WF*_NiO_ = 4.0 eV. Here shall be addressed that PMMA, being an amorphous polymeric material and lacking a conventional valence band as those in crystalline solids, is better described in using its ionization energy. This value reflects the energy of the highest occupied molecular orbital (HOMO), rather than relying on the concept of a valence band. The valence band maximum edge (*E_V_*) was then derived using *E_V_* = *WF* + *E*_VBM_. The calculated *E_V_* values for ZnO, CsPbBr_3_ QDs, and NiO are 7.30 eV, 5.91 eV, and 5.10 eV, respectively. Similarly, the work functions of the device’s conductive materials, including Ag, SLG, and Au electrodes, were determined as *WF*_Ag_ = 4.36 eV, *WF*_SLG_ = 4.30 eV, and *WF*_Au_= 5.12 eV, respectively. On the other hand, LEIPS was utilized to probe the unoccupied electronic states (conduction bands, *E_C_*) of the materials, enabling direct measurement of electron affinity (*E*_A_, *qc*). Based on the LEIPS spectra (see insets of **Figure S2a**), the conduction band minimum edge (*E_C_*) were determined to be 3.60 eV, 1.80 eV, 3.50 eV, and 1.85 eV for ZnO, PMMA, CsPbBr_3_ QDs, and NiO, respectively. Combining UPS and LEIPS data allowed us to calculate the energy gaps (*E*_g_) of the materials, which are *E_g_* = 3.70 eV, 5.70 eV, 2.41eV, and 3.25 eV, for ZnO, PMMA, CsPbBr_3_ QDs, and NiO, respectively. By combining the energy band structures of all the materials, we can construct a comprehensive energy-band diagram of the TP photodetector, as illustrated in **Figure S2b** and **S2c**, which depict the states before and after thermal equilibrium. The dashed line in these diagrams indicates the Fermi level. Accordingly, ZnO and CsPbBr_3_ QDs exhibit n-type polarity, while NiO exhibits p-type polarity. Additionally, the high work functions of Au and Ag enable them to form direct ohmic contacts with their respective adjacent layers, SLG and ZnO, which facilitates the efficient collection of photogenerated carriers.

**Supplementary Figure 3**

**
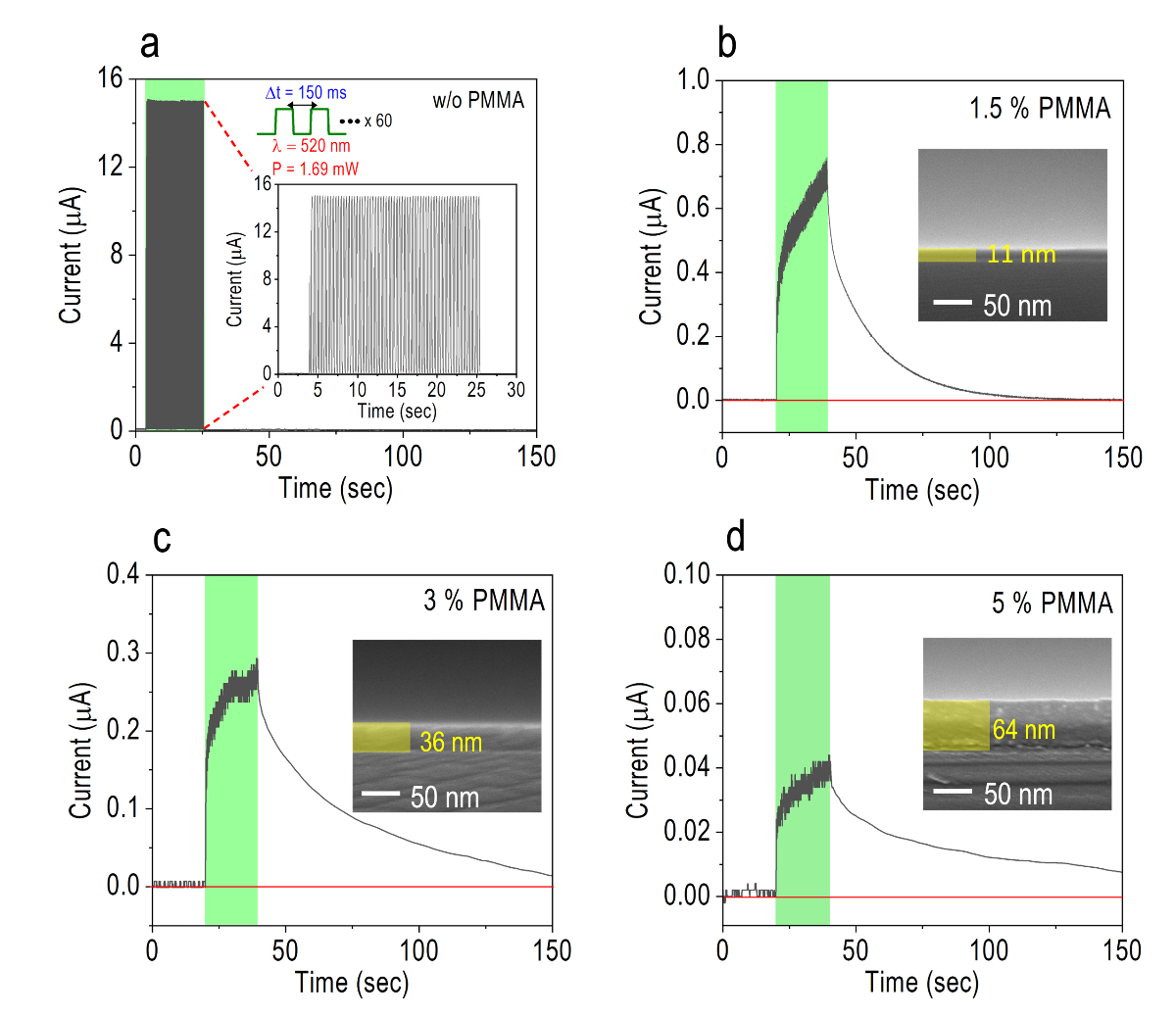
**

**Figure S3**. *EPSC* responses of TP photodetector devices. **a)**  A control device fabricated without a PMMA interlayer, alongside devices incorporating PMMA interlayers with varying thicknesses of **b)** 11 nm (1.5 wt%), **c)** 36 nm (3.0 wt%), and **d)** 64 nm (5.0 wt%). Synaptic potentiation in each device is induced by a series of 60 consecutive optical stimuli ($\lambda=$ 520 nm, *P* = 1.69 mW, $t_{p}=$ 150 ms, and $\Delta t$ = 150 ms, as depicted in the inset of **Figure** **S3a**), as denoted by the green-shaded region. An enlarged view focusing on the region subjected to repeated optical stimuli is also inserted in **Figure** **S3a**. Corresponding cross-sectional SEM images of the PMMA interlayers are also inserted in **Figures** **S3b**, **S3c** and **S3d**.

**Figure** **S3** presents the *EPSC* responses of **a)** the control device without a PMMA interlayer, as well as those of TP photodetector devices incorporating PMMA interlayers with varying thicknesses of **b)** 11 nm (1.5 wt%), **c)** 36 nm (3.0 wt%), and **d)** 64 nm (5.0 wt%). The control device exhibits no apparent synaptic potentiation under repeated optical stimuli, underscoring the essential role of the PMMA interlayer in enabling synaptic functionalities. The insertion of the PMMA interlayer introduces a discontinuity at $E_{c}$, which effectively promotes the trapping and accumulation of photogenerated electrons (**Figure** **1d**). This charge accumulation modulates the current conduction, which is a prerequisite for emulating synaptic behavior. Following optical stimulation, the *EPSC* responses gradually decay in the absence of an external electrical bias, reflecting the devices’ intrinsic relaxation dynamics. Notably, devices with thicker PMMA interlayers exhibit prolonged *EPSC* retention times, indicative of enhanced memory retention capabilities. However, this enhancement is accompanied by a decrease in *EPSC* amplitude, mainly due to the increased series resistance associated with the thicker PMMA interlayers. This observation points to a fundamental trade-off between retention time and photocurrent extraction efficiency. Based on these findings, a PMMA concentration of 1.5 wt% was selected for this study, as it offers an optimal balance between photoresponsivity and synaptic retention, both of which are essential for the effective emulation of biological synaptic functionalities.

**Supplementary Figure 4**


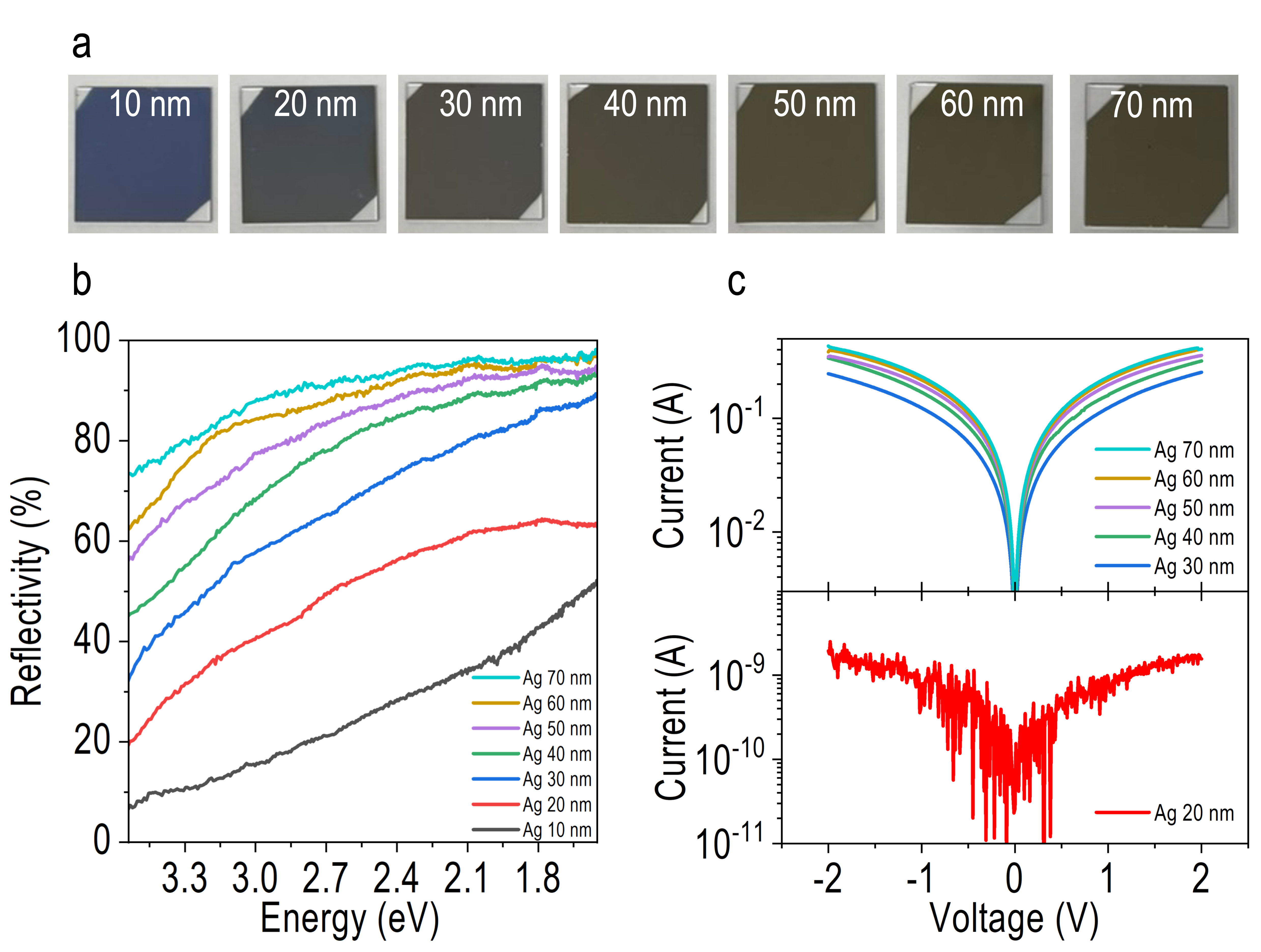


**Figure S4** **a)** Photographs of Ag films deposited on glass substrates with thicknesses ranging from 10 nm to 70 nm. **b)** Reflectivity spectra and **c)** *I-V* characteristics of Ag films of various thicknesses.

In the TP photodetector, the Ag thin film deposited on the device provides a dua function: acting as a reflective mirror and as the Ohmic contact electrode. Selecting the optimal Ag thickness is important to effectively excite the TP eigenmode while ensuring sufficient electrical conductivity. **Figure** **S4a** shows photographs of Ag films deposited on glass substrates with thicknesses ranging from 10 nm to 70 nm. As the thickness increases from 10 nm to 30 nm, the films change color from bluish-purple to metallic gray, indicating significant shifts in their reflectivity spectra. Beyond 30 nm, the Ag films stabilize into a consistent opaque metallic gray, implying a gradual saturation in their reflectivity. **Figure** **S4b** displays the reflectivity spectra of Ag films of various thicknesses. Thicker Ag films enhance reflectivity and hence improve the optical quality of the TP cavity; however, they also reduce the number of incident photons available for detection, which creates a trade-off between Ag thickness and photodetection performance. The *I-V* characteristics of Ag films with thicknesses ranging from 20 nm to 70 nm are plotted in **Figure** **S4c**, where the 10 nm film is excluded due to its current being below the detection limit of the source meter (Keithley 2400). The 20 nm Ag film exhibits low conductivity, with current values measured ranging from 0.1–1.0 nA, rendering it unsuitable as an electrode. In contrast, Ag films thicker than 30 nm demonstrate consistent and stable electrical performance, with current values ranging from 0.01–0.1 A, making them ideal for electrode applications. For this study, a 40 nm Ag film was chosen as the optimal thickness, offering a balance between high reflectivity of exciting TP eigenmode and sufficient electrical conductivity.

**Supplementary Figure 5**

*
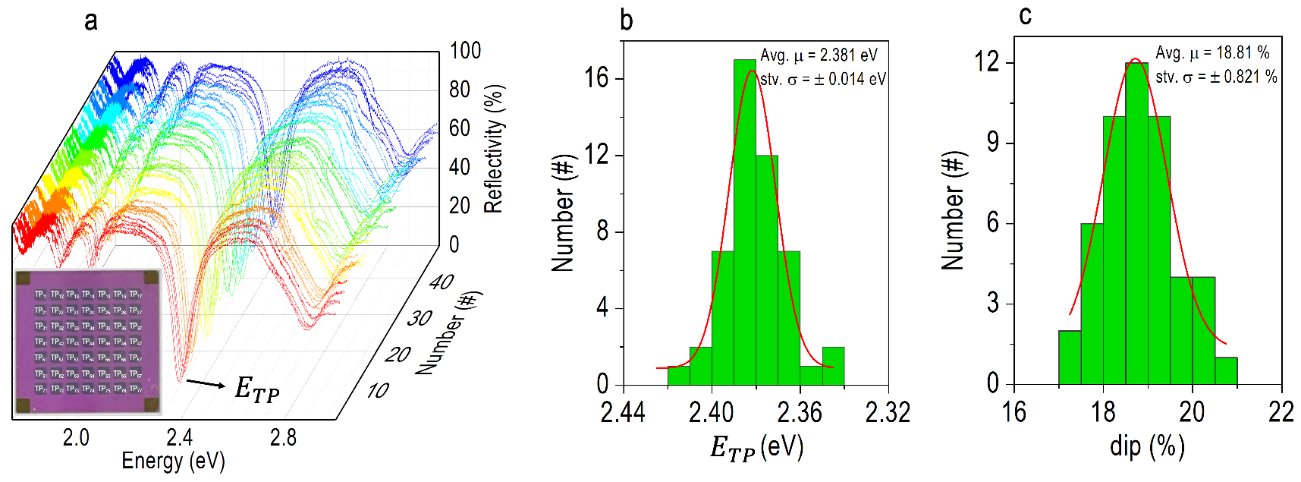
*

**Figure S5 a)** Reflectance spectra of the $7\times7$ CsPbBr_3_ QD-based TP photodetector array. An optical image of the array is inserted in the figure, with each individual device labeled according to its corresponding position within the array. Statistical distributions of the cumulative counts as functions of **b)** the resonant energy ($E_{TP}$) and **c)** the reflectance dip ($R_{dip}$) for the full TP photodetector array.

**Figure** **S5a** shows the reflectance spectra obtained from 49 devices of the CsPbBr_3_ QD-based TP photodetectors array, aimed at evaluating the extent of device-to-device variability in the resonant energy of the TP eigenmode ($E_{TP})$and its associated reflectivity dip ($R_{dip})$. As illustrated in **Figure** **S5b**, the distribution of the resonant energy of the TP photodetector is centered at a mean value of $E_{TP}=2.381 eV$, with a relatively small standard deviation of $\sigma=\pm0.014$ $eV$, indicating a high degree of uniformity across devices. In parallel, the reflectivity dip at $E_{TP}$demonstrates a mean value of $R_{dip}=18.81\%$, accompanied by a slightly larger standard deviation of $\sigma=\pm0.821\%$, as shown in **Figure S5c**. The above observations validate that our CsPbBr_3_ QD-based TP photodetector exhibits an acceptable device-to-device variability.

**Supplementary Figure 6**


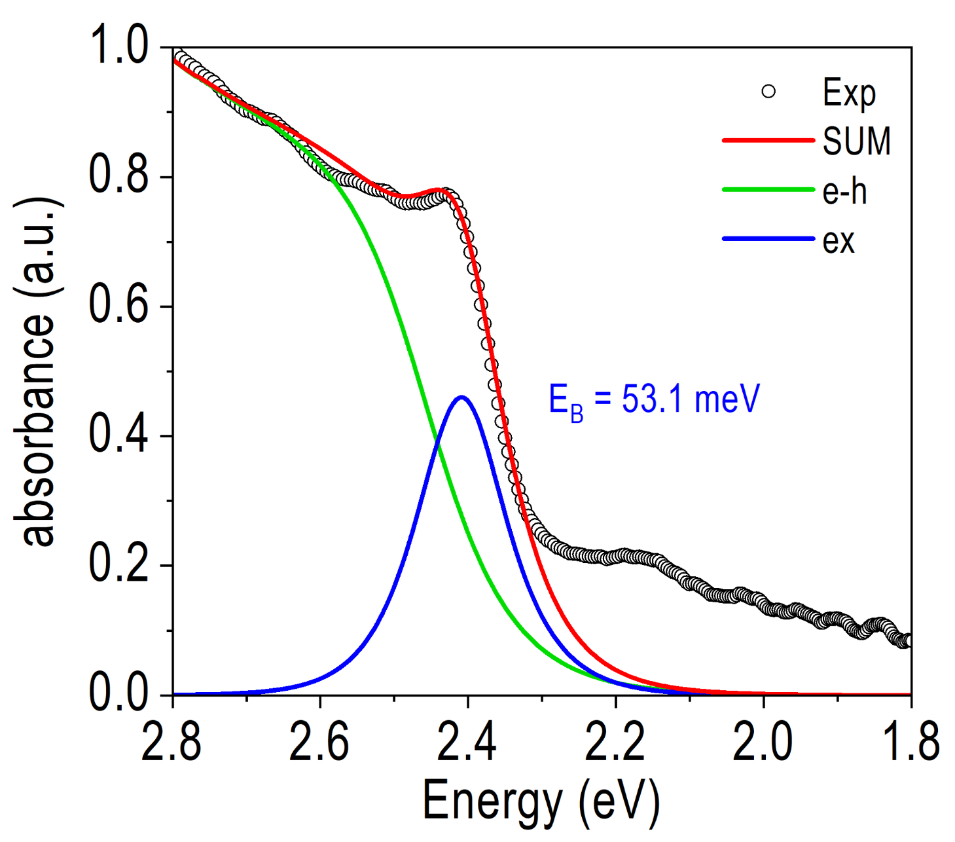


**Figure S6**. Absorption spectrum of the CsPbBr_3_ QDs (black open circles) along with the theoretical fit (red line). Individual contributions from excitonic (blue line) and band-to-band (green line) transitions are estimated according to Elliot’s theory of Wannier excitons.

In this study, efficient coupling between incident photons and QDs' excitons significantly affects the energy absorption of the TP photodetector. The exciton binding energy (*E_B_*) of CsPbBr_3_ QDs is an important parameter for assessing the photon-exciton coupling strength. To determine the *E_B_* of the synthesized CsPbBr_3_ QDs, we measured the absorption spectrum at room temperature and modeled it using Elliott’s theory ^1,2^, which accounts for hydrogen-like excitonic effects, as shown in **Figure S6**. A strong agreement between the theoretical model and the experimental data was achieved. In the resulting Elliott fit (red curve), the exciton peak (blue line) and the continuous interband absorption (green line) are clearly distinguishable, yielding an *E_B_* of 53.1 meV. This value, which is significantly higher than the thermal energy at room temperature (25.6 meV), is beneficial to enhancing photon-exciton coupling under ambient conditions.

1. M. Saba, M. Cadelano, D. Marongiu, *Nat. Commun.* **2014**, 5, 5049.
2. M. Baranowski, P. Plochocka, *Adv. Energy Mater.* **2020**, 10, 1903659.

**Supplementary Figure 7**

*
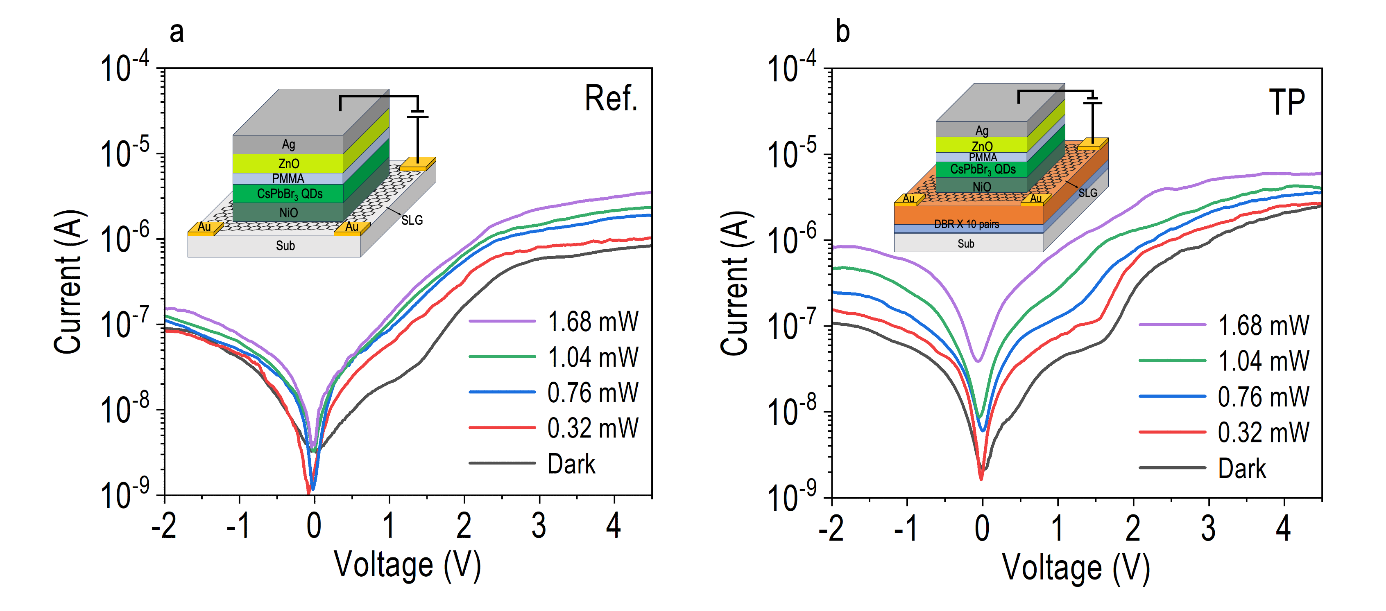
*

**Figure S7** Semi-logarithmic *I-V* characteristics of **a)** the reference device and **b)** the TP photodetector under green-light illumination ($\lambda=$ 520 nm) at varying optical power levels ranging from 0.32 to 1.68 mW. Schematic illustrations depicting the forward bias applied across the devices are included in both figures for clarity.

To further examine the rectifying characteristics of the fabricated photodetector devices, the linear *I-V* characteristics previously presented in **Figure** **5a** are re-plotted on a semi-logarithmic scale [log(I)] in **Figure** **S7**, where the electrical measurements are conducted under an applied bias ranging from $-2$ to $+4.5$ for both **a)** the reference device and **b)** the TP photodetector, across a range of optical power from 0.32 to 1.68 mW. Irrespective of illumination conditions, both devices exhibit clear rectifying behaviors with a consistent turn-on voltage in the range of approximately 2.3 to 2.6 V, suggesting a well-defined p-n junction intrinsically formed within the device structure.

**Supplementary Figure 8**


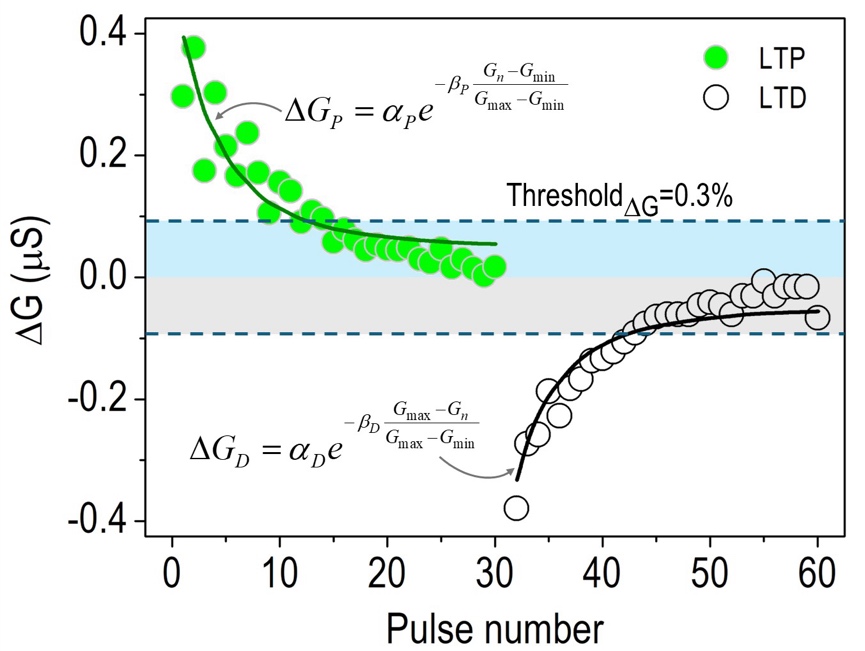


**Figure S8**. The variation of *∆G* as a function of the number of applied pulses to the TP photodetector, inclusive of both the LTP (green dots) and LTD (open dots) processes. In this figure, the LTP process is induced by applying 30 consecutive light pulses under on-resonance illumination condition ($\lambda=520 nm)$, while the LTD process is triggered by 30 consecutive negative electrical pulses to the TP photodetector.

**Figure S8** illustrates the consecutive conductance variation (*∆G*) as a function of the number of applied pulses to the TP photodetector, demonstrating both the LTP and LTD processes. In synaptic devices, the parameter $\beta$ quantifies the degree of nonlinearity in conductance ($G$) relative to external stimuli, while the number of effective conductance states ($N_{eff}$) measures the extent to which the synaptic device can be modulated linearly by these stimuli. Both $\beta$ and $N_{eff}$ are key indicators of the complex response to external inputs. Typically, a lower $\beta$ value corresponds to a higher $N_{eff}$, which enhances the device's ability to modulate synaptic weights effectively, thereby improving computational accuracy and learning performance in neural networks.

In this study, we performed a nonlinear analysis on our TP photodetector, utilizing fitting parameters derived from the weight update equations below:

$G_{n+1}=G_{n}+\Delta G_{P}=G_{n}+\alpha_{P}e^{-\beta_{P}\frac{G_{n}-G_{min}}{G_{max}-G_{min}}}$ (S3)

$G_{n+1}=G_{n}+\Delta G_{D}=G_{n}-\alpha_{D}e^{-\beta_{D}\frac{G_{max}-G_{n}}{G_{max}-G_{min}}}$ (S4)

Here $G_{n+1}$and $G_{n}$ represent the device’s conductance at the (${n+1)}^{th}$ and $n^{th}$ pulse, respectively, while $G_{max}$ and $G_{min}$ denote the maximum and minimum conductance values. Parameters $\alpha_{P}$ (potentiation) and $\alpha_{D}$ (depression) indicate the step sizes of the conductance changes, whereas $\beta_{P}$ and $\beta_{D}$ are the nonlinearity factors for the LTP and LTD synaptic processes. From **Figure S8**, we extracted the nonlinear factors of our TP photodetector, finding that $\beta_{P}=\beta_{D}\approx$2. The parameter $N_{eff}$ is defined as the number of instances where $\left| \Delta G_{P, D} \right|$ exceeds 3.0% of $\left| G_{max}-G_{min} \right|$, a threshold set to exclude minor conductance variations that are insignificant for neuromorphic computing. This exclusion reduces computational power consumption. For instance, in **Figure S8**, among 30 ∆G points during the LTP process, 15 values fall below the threshold, resulting in *N*_eff_ being 15 out of 30. Following the same approach, the number of effective conductance states for the LTD process also yields $N_{eff}=\text{15}$.

**Supplementary Figure 9**


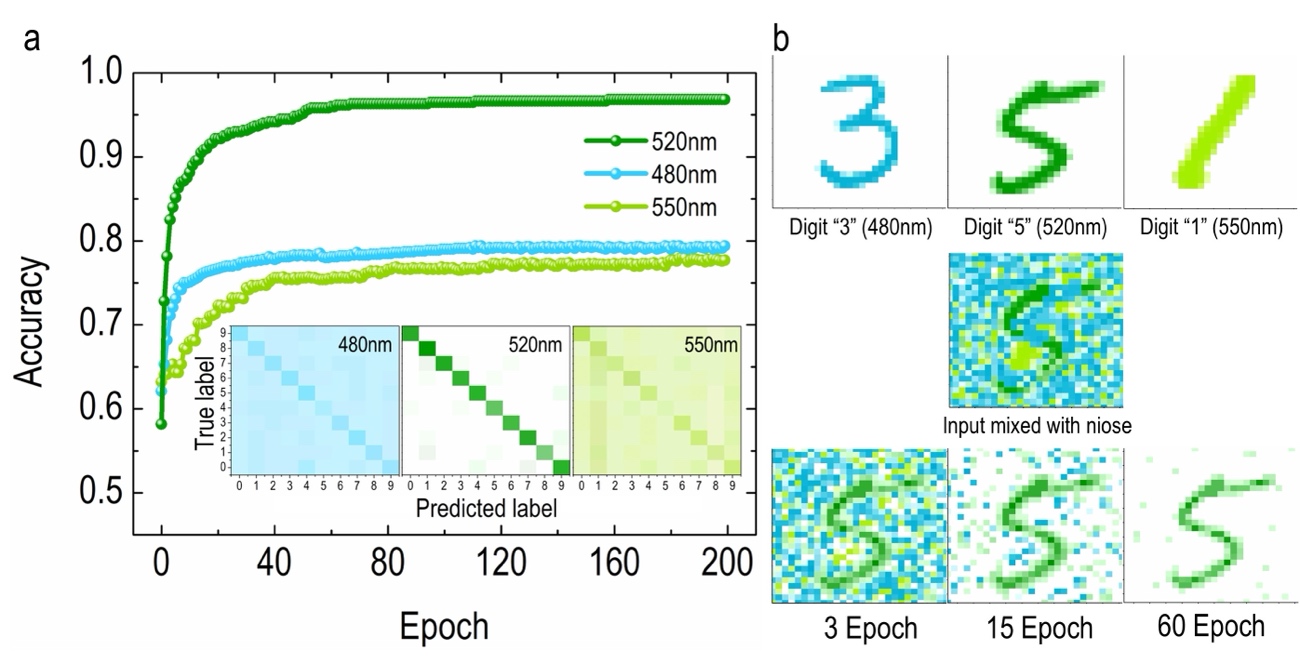


**Figure S9 a)** Recognition accuracy of color-specific ($\lambda=480/520/550 nm$ ) MNIST handwritten digit datasets as a function of the number of training epochs. Insets: confusion matrices of classification results for the identical digit datasets after 200 training epochs. **b)** An encoded image (center) comprising MNIST handwritten numerals mixed with different colors, including digits 5 ( $\lambda=520$ nm), 3 ( $\lambda=480$ nm), and 1 ( $\lambda=550$ nm), was input into a pre-trained neural network to demonstrate wavelength-selective recognition capability of the TP device.

When the energy of the incident photons matches the resonant energy of the TP device, strong light–matter interactions within the CsPbBr_3_ QDs are induced, resulting in significantly enhanced optical absorption and elevated photocarrier generation. The increased carrier population contributes to a more efficient carrier injection across the interfacial potential barrier, thereby intensifying the synaptic response. Under resonant illumination ($\lambda=520 nm$), the TP device consequently exhibits superior synaptic performance, characterized by a reduced nonlinearity parameter and an increased number of effective conductance states, as compared to those observed under off-resonance illumination conditions ($\lambda=480 \mathrm{or} 550 nm)$. Specifically, under resonant excitation, the estimated values of nonlinearity and effective conductance states are $\beta_{P}\approx$2 and$N_{eff}=15$, respectively (refer to **Figure S8**), whereas the off-resonance illimitation condition yields $\beta_{P}$ values in the range of approximately $3.5-3.7$ and$N_{eff}$ values between 8 and 9.

To further validate the resonance-enhanced synaptic performance of the TP device, we assessed its image recognition accuracy utilizing color-specific ($\lambda=480/520/550 nm$ ) MNIST handwritten digit datasets (from digits “0” to “9”), as illustrated in **Figure S9a**. Since the digit dataset with the color hue at $\lambda=520$ nm, aligns with the resonant energy of the TP device, it elicits a significantly enhanced synaptic response. This resonance coupling manifests in markedly superior recognition accuracy compared to the other two datasets with color hues at off-resonant wavelengths of $\lambda=480$ and $550$ nm. Specifically, the image recognition accuracy for the $\lambda=520$ nm dataset exhibits a rapid increase, reaching 90% within merely 15 training epochs, clearly outperforming the accuracies achieved by $\lambda=480$ nm (76%) and $\lambda=550$ nm (70%) under the same training epoch condition. The inserted confusion matrices further corroborate this observation, which demonstrates a more pronounced diagonal contrast in the $\lambda=520$ nm dataset (middle), indicative of superior classification accuracy than $\lambda=480$ nm (left) and $\lambda=550$ nm (right) datasets.

Finally, to assess the feasibility of implementing color-mixed image recognition using the TP device, an encoded image comprising MNIST handwritten numerals mixed with different colors, including digits 5 ( $\lambda=520$ nm), 3 ( $\lambda=480$ nm), and 1 ( $\lambda=550$ nm), was input into a pre-trained neural network, as shown in **Figure S9b** where deliberate background noise has been incorporated into the image to enhance complexity. During this process, the encoded image was subjected to classification based on its color-specific features through optimized synaptic weight allocation. Owing to the distinct synaptic weight modulation in response to resonant and off-resonance optical stimuli, along with the progressive increase in training epochs, the neural network integrated with the TP devices can successfully distinguish the encoded digits. Ultimately, only the digit corresponding to the resonant wavelength of the TP device ( $\lambda=520$ nm) remained prominent after 60 training epochs, demonstrating its superior wavelength-selective recognition capability.

**Supplementary Note S1**

Here, we illustrate detailed derivations of how to quantitatively evaluate the photoresponse performance of the TP photodetector in this work, including its responsivity ($R_{\lambda}$) and detectivity ($D^{*}$). Responsivity measures the efficiency of the photodetector in converting incident light into photocurrent. It is defined as the ratio of the generated photocurrent ($I_{ph}$) to incident light power ($P$), as described by the following equation:

$R_{\lambda}=\frac{I_{ph}}{P}=\frac{(I_{i\mathrm{illumination}}-I_{dark})}{P}$ (S5)

where $I_{i\mathrm{illumination}}$ and $I_{dark}$ represent the photocurrent under illumination and the dark current, respectively.

Detectivity quantifies the minimum level of incident light that the photodetector can reliably detect. It is heavily influenced by the dark current and is expressed as:

$D^{*}=\frac{R_{\lambda}}{\sqrt{2qJ_{dark}}}$ (S6)

where $J_{dark}$ is the dark current density and q is the elementary charge. As the incident light power increases, $D^{*}$exhibits a similar decreasing trend. This behavior is primarily driven by the responsivity ($R_{\lambda}$) in the numerator of **Equation S6**, along with the consistently low dark current observed in the TP photodetector.

**Supplementary Note S2**

To enhance facial feature recognition and reduce redundant information, we have implemented an ANN-based weight-update expectation thresholding model, a well-established technique in computer vision. This model transforms continuous image data into a discrete format, enabling effective segmentation of key features from the background. It follows the traditional structure of an ANN, beginning with backpropagation, where the gradient of the loss function $\nabla L\left( w \right)={\partial L}/{\partial w}$is computed (with $L$ and $w$ representing the loss function and the synaptic weights, respectively). This gradient is then applied in the gradient descent process, as described by the following equation:

$w^{(n+1)}=w^{(n)}-\eta\frac{\partial L}{\partial w}$ (S7)

where $w^{(n+1)}$represents the updated weight after iteration $n+1$, $\eta$ is the learning rate, and ${\partial L}/{\partial w}$ is the gradient obtained from backpropagation. Through this iterative process, the synaptic weights are adjusted to minimize the error between predicted and true labels. This allows us to create a conductance mapping for the input images to conduct facial recognition. In this work, the intensity of conductance mapping varies within $\pm\left( 0.98-3.33 \right)\times{10}^{-7}S$, largely influenced by LTP and LTD processes in the TP photodetector. Finally, a threshold is applied by selecting the top 32% of the conductance values from each synaptic device. This thresholding model effectively activates the most significant pixels, emphasizing key facial features in the input images for better facial recognition.

**Table *I***


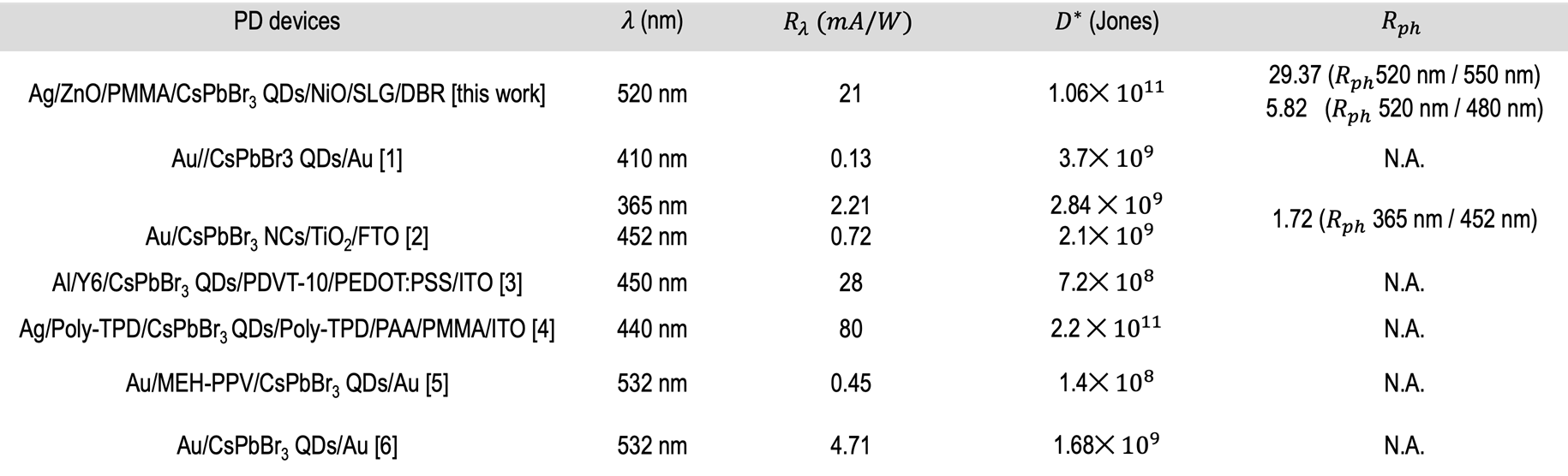


**Table *I*** Comparison of key photoresponse performance metrics, including responsivity ($R_{\lambda}$), detectivity ($D^{*}$), and photocurrent selectivity ($R_{\mathrm{ph}}$), between our proposed TP photodetector and previously reported CsPbBr_3_ QD-based photodetectors.

1. A. Ghosh, S. Paul, M. Das, P. K. Sarkar, P. Bhardwaj, G. Sheet, and S. Acharya, *ACS Nano* **2024**, 18, 23310.
2. A. Suhail, A. Saini, S. Beniwal, M. Bag, *J. Phys. Chem. C* **2023**, *127*(34), 17298–17306.
3. K. Chen, X. Zhang, P. A. Chen, J. Guo, M. He, Y. Chen, Y. Hu, *Adv. Sci.* **2022**, *9*(12), 2105856.
4. H. Algadi, C. Mahata, B. Sahoo, M. Kim, W. G. Koh, T. Lee, *Org. Electron.* **2020**, *76*, 105444.
5. L. Jin, Y. Zhang, M. Cao, Y. Yu, Z. Chen, Y. Li, J.Yao, *Nanotechnology* **2021**, *32*(23), 235203.
6. Y. Dong, Y. Gu, Y. Zou, J. Song, L. Xu, J. Li, H. Zeng, *Small* **2016**, *12*(40), 5622–5632.
